# Selection of an HLA-C*03:04-Restricted HIV-1 p24 Gag Sequence Variant Is Associated with Viral Escape from KIR2DL3+ Natural Killer Cells: Data from an Observational Cohort in South Africa

**DOI:** 10.1371/journal.pmed.1001900

**Published:** 2015-11-17

**Authors:** Angelique Hölzemer, Christina F. Thobakgale, Camilo A. Jimenez Cruz, Wilfredo F. Garcia-Beltran, Jonathan M. Carlson, Nienke H. van Teijlingen, Jaclyn K. Mann, Manjeetha Jaggernath, Seung-gu Kang, Christian Körner, Amy W. Chung, Jamie L. Schafer, David T. Evans, Galit Alter, Bruce D. Walker, Philip J. Goulder, Mary Carrington, Pia Hartmann, Thomas Pertel, Ruhong Zhou, Thumbi Ndung’u, Marcus Altfeld

**Affiliations:** 1 Ragon Institute of MGH, MIT and Harvard, Cambridge, Massachusetts, United States of America; 2 Heinrich-Pette-Institut, Leibniz Institute for Experimental Virology, Hamburg, Germany; 3 First Department of Internal Medicine, University Medical Center Hamburg—Eppendorf, Hamburg, Germany; 4 HIV Pathogenesis Programme, Doris Duke Medical Research Institute, KwaZulu-Natal Research Institute for Tuberculosis and HIV, Nelson R. Mandela School of Medicine, University of KwaZulu-Natal, Durban, South Africa; 5 Computational Biology Center, IBM Thomas J. Watson Research Center, Yorktown Heights, New York, United States of America; 6 Microsoft Research, Los Angeles, California, United States of America; 7 Experimental Immunology, Academic Medical Center, Amsterdam, The Netherlands; 8 Department of Microbiology and Immunobiology, Harvard Medical School, Boston, Massachusetts, United States of America; 9 Division of Microbiology, New England Primate Research Center, Southborough, Massachusetts, United States of America; 10 Department of Pathology and Laboratory Medicine, University of Wisconsin, Madison, Madison, Wisconsin, United States of America; 11 Department of Paediatrics, University of Oxford, Oxford, United Kingdom; 12 Cancer and Inflammation Program, Laboratory of Experimental Immunology, Leidos Biomedical Research, Frederick National Laboratory for Cancer Research, Frederick, Maryland, United States of America; 13 First Department of Internal Medicine, Division of Infectious Diseases, University of Cologne, Cologne, Germany; 14 Institute for Medical Microbiology, Immunology and Hygiene, University of Cologne, Cologne, Germany; 15 Center for Neurologic Diseases, Brigham and Women’s Hospital and Harvard Medical School, Boston, Massachusetts, United States of America; 16 Max Planck Institute for Infection Biology, Berlin, Germany; 17 KwaZulu-Natal Research Institute for Tuberculosis and HIV, Nelson R. Mandela School of Medicine, University of KwaZulu-Natal, Durban, South Africa; St. Vincent's Hospital, AUSTRALIA

## Abstract

**Background:**

Viruses can evade immune surveillance, but the underlying mechanisms are insufficiently understood. Here, we sought to understand the mechanisms by which natural killer (NK) cells recognize HIV-1-infected cells and how this virus can evade NK-cell-mediated immune pressure.

**Methods and Findings:**

Two sequence mutations in p24 Gag associated with the presence of specific *KIR/HLA* combined genotypes were identified in HIV-1 clade C viruses from a large cohort of infected, untreated individuals in South Africa (*n* = 392), suggesting viral escape from KIR+ NK cells through sequence variations within HLA class I—presented epitopes. One sequence polymorphism at position 303 of p24 Gag (T_Gag303_V), selected for in infected individuals with both *KIR2DL3* and HLA-C*03:*04*, enabled significantly better binding of the inhibitory KIR2DL3 receptor to HLA-C*03:04-expressing cells presenting this variant epitope compared to the wild-type epitope (wild-type mean 18.01 ± 10.45 standard deviation [SD] and variant mean 44.67 ± 14.42 SD, *p* = 0.002). Furthermore, activation of primary KIR2DL3+ NK cells from healthy donors in response to HLA-C*03:04+ target cells presenting the variant epitope was significantly reduced in comparison to cells presenting the wild-type sequence (wild-type mean 0.78 ± 0.07 standard error of the mean [SEM] and variant mean 0.63 ± 0.07 SEM, *p* = 0.012). Structural modeling and surface plasmon resonance of KIR/peptide/HLA interactions in the context of the different viral sequence variants studied supported these results. Future studies will be needed to assess processing and antigen presentation of the investigated HIV-1 epitope in natural infection, and the consequences for viral control.

**Conclusions:**

These data provide novel insights into how viruses can evade NK cell immunity through the selection of mutations in HLA-presented epitopes that enhance binding to inhibitory NK cell receptors. Better understanding of the mechanisms by which HIV-1 evades NK-cell-mediated immune pressure and the functional validation of a structural modeling approach will facilitate the development of novel targeted immune interventions to harness the antiviral activities of NK cells.

## Abstract 2

An analysis from a cohort in South Africa reveals how the HIV virus may escape NK cell immunity by acquiring mutations in HLA-mediated epitopes, which affect binding to NK cell receptors.

## Editors' Summary

**Background:**

Throughout life, our immune system—a complex network of cells, tissues, and organs—protects us from attack by viruses, bacteria, parasites, and fungi. The body’s first line of defense against these “pathogens” is the innate immune system, a collection of cells and proteins that is always ready to identify and kill a wide range of foreign invaders. As well as directly killing pathogens, the innate immune system activates the adaptive immune response, which recognizes and kills specific pathogens and is responsible for immunological memory. Most pathogens are dispatched quickly and effectively by the two arms of the immune system, but some infectious agents have found ways to evade the immune response. For example, infection with HIV-1, the virus that causes AIDS, results in prolonged, continuous viral replication even though the human body mounts a vigorous HIV-1-specific immune response. In large part, HIV-1’s evasion of the immune response reflects its ability to kill virus-specific CD4 lymphocytes, which are needed to help other immune system cells kill HIV-1-infected cells. In addition, the proteins on the surface of HIV-1 that are recognized by the human immune system (viral antigens) frequently acquire changes (mutations) that make it harder for the immune system to clear HIV-1 from the human body.

**Why Was This Study Done?:**

Viruses evade immune surveillance in many ways, and if we understood the mechanisms underlying immune evasion better, it might be possible to develop targeted immune interventions to deal with viruses such as HIV-1. Here, the researchers investigate how natural killer (NK) cells, a type of lymphocyte that is an important component of the innate antiviral immune response, recognize HIV-infected cells and how HIV-1 evades NK-cell-mediated immune pressure. NK cell activation is determined by the integration of inhibitory and activating signals delivered to the cells by several different receptor families, including the family of killer-cell immunoglobulin-like receptors (KIRs). KIRs mainly bind to human leukocyte antigen (HLA) class I molecules (ligands) on their target cells. HLA class I proteins display fragments (epitopes; peptides recognized by the immune system) of pathogens present in infected cells on the cell surface so that the immune system knows that that cell needs destroying. The binding of distinct KIRs to HLA class I ligands depends on both the sequence of the HLA class I molecule and the sequence of the epitope presented by that HLA class I molecule. Thus, the researchers hypothesized that HIV-1 might evade NK-cell-mediated immune surveillance by acquiring mutations within epitopes presented by HLA class I molecules that enhance the engagement of inhibitory KIRs on NK cells, thereby inhibiting NK cell activity.

**What Did the Researchers Do and Find?:**

To investigate this model, the researchers asked whether any polymorphisms (naturally occurring genetic variations) in the HIV-1 gene encoding the p24 Gag protein were selected on a population level in HIV-1-infected individuals expressing specific combinations of KIRs and HLA class I ligands. Using statistical methods to identify *KIR/HLA* combined genotypes in a large group of untreated HIV-1-infected individuals from South Africa, they showed that a specific sequence polymorphism in p24 Gag was selected for in individuals expressing both HLA-C*03:*04* and *KIR2DL3*. Functional studies showed that the selection of this variant HIV-1 epitope resulted in better binding of KIR2DL3, an inhibitory KIR, to HLA-C*03:04 than the wild-type epitope. Moreover, the activation of KIR2DL3-positive NK cells from healthy donors in response to HLA-C*03:04-positive target cells presenting the variant epitope was significantly reduced compared to the activation of KIR2DL3-positive NK cells in response to target cells presenting the wild-type epitope.

**What Do These Findings Mean?:**

Further studies are needed to assess the consequences of this and other viral sequence variants for viral fitness, the processing and presentation of the mutant epitope during natural infections, and the control of HIV-1 replication in patients. However, these findings provide new insights into how HIV-1 (and possibly other viruses that have a high mutation rate) might evade NK cell immunity through the selection of mutations in HLA-presented epitopes that enhance the binding of inhibitory KIRs to HLA class I/peptide complexes. A better understanding of this molecular mechanism for evasion of immune surveillance should facilitate the development of targeted immune interventions (for example, the use of KIR-blocking antibodies, some of which are already being clinically tested for the treatment of cancer) to maximize the antiviral activities of NK cells.

**Additional Information:**

This list of resources contains links that can be accessed when viewing the PDF on a device or via the online version of the article at http://dx.doi.org/10.1371/journal.pmed.1001900.

- The US National Institute of Allergy and Infectious Diseases provides a simple description of the human immune system and information on all aspects of HIV infection and AIDS
- Information is available from Avert, an international AIDS charity, on many aspects of HIV/AIDS; Avert also provides personal stories about living with HIV/AIDS
- The British Society for Immunology provides short articles about various aspects of immunology, including general information about host–pathogen interactions and immune evasion and specific information about HIV and immune evasion
- Wikipedia has pages on natural killer cells, KIRs, and HLA molecules (note that Wikipedia is a free online encyclopedia that anyone can edit; available in several languages)

## Introduction

Natural killer (NK) cells are an important component of the antiviral innate immune response. They have the ability to lyse target cells without prior antigen sensitization and to regulate adaptive immune responses by secreting chemokines and cytokines [1]. NK cell activation is determined by the integration of inhibitory and activating signals delivered by a number of different receptor families, including the killer-cell immunoglobulin-like receptors (KIRs), which predominantly recognize human leukocyte antigen (HLA) class I ligands [2]. The binding of distinct KIRs to their HLA class I ligands on target cells is determined not only by conserved motifs within the α1 and α2 helixes of the HLA class I molecule but also by the sequence of the peptide presented by the respective HLA class I molecule [3–8]. The important role of the sequence of the HLA-presented peptide has been further emphasized by the recent resolution of crystal structures of KIR/peptide/HLA complexes, showing that the engagement of several inhibitory KIRs, including KIR3DL1 and KIR2DL2, is highly susceptible to changes in the carboxyl-terminal residues of the HLA class I—presented peptide [9,10].

Interactions between KIR and HLA class I ligands have been shown to play an important role in the outcome of viral infection [11]. Several epidemiological studies have demonstrated a protective role of specific KIR/HLA combined genotypes in HIV-1 disease outcome. HIV-1-infected individuals with *KIR3DS1* and *HLA-B* alleles of the HLA-Bw4 family, with an isoleucine at position 80 exhibit a significantly slower progression to AIDS [12], and certain alleles of *KIR3DL1* resulting in high surface expression of KIR3DL1 are associated with better control of HIV-1 viremia in individuals with *HLA-Bw4* [13]. HIV-1 transmission in HLA-discordant couples was suggested to be reduced in *KIR2DL2/3+* individuals [14]. Furthermore, single nucleotide polymorphisms associated with higher expression of HLA-C molecules that serve as ligands for KIR2DL1/2/3 have also been associated with better control of HIV-1 infection [15]. The precise mechanisms by which these KIR/HLA interactions can modulate the outcome of HIV-1 infection are not well understood, but increasing data suggest a role for KIR-expressing NK cells in mediating antiviral activity [16–24].

Viruses have evolved multiple mechanisms to evade antiviral immune responses. HIV-1 escape from virus-specific CD8+ T cell recognition through the selection of single amino acid mutations in targeted epitopes has been well established, and can lead to impairment of immune-mediated viral control [25–27]. Similarly, KIR-associated sequence polymorphisms within HIV-1 might allow for viral escape from NK-cell-mediated immune recognition [28]. However, the mechanisms by which sequence polymorphisms within HIV-1 can enable evasion from antiviral NK cells are not understood. One possible mechanism is that viral sequence mutations within HLA class I—presented epitopes might lead to enhanced engagement of inhibitory KIRs expressed on NK cells and thereby inhibit NK cell activity against infected cells. Here we provide novel data supporting this model in a large cohort of individuals from Durban, South Africa, infected with HIV-1 clade C, demonstrating, to our knowledge for the first time, that a combined *KIR/HLA*-associated sequence polymorphism within p24 Gag selected in individuals with a certain *KIR/HLA* genotype is associated with escape from recognition by the respective KIR+ NK cells.

## Methods

### Ethics Statement

Written informed consent was acquired from all study participants enrolled in the Sinikithemba cohort, and the study protocol was approved by the Biomedical Research Ethics Committee of the University of KwaZulu-Natal (approval number E028/99). The study of primary NK cell function in nine healthy HIV-1-negative individuals recruited at Massachusetts General Hospital (Boston, MA) and at Brigham and Women’s Hospital (Boston, MA; IGTB cohort) was approved by the Partners Human Research Committee (protocol #2012P002121), and each participant gave written informed consent for participation.

### Study Participants

Study participants were recruited from the Sinikithemba cohort in Durban, South Africa, and included 406 individuals chronically infected with HIV-1 clade C. The study participants were antiretroviral-naïve, KIR typed, and HLA typed to four-digit resolution by molecular methods [29]. Viral load and CD4 T cell measurements at study entry were obtained using the Roche Amplicor assay (version 1.5) and TruCount technology, respectively. At baseline the median age of the cohort was 31 y (interquartile range [IQR] 27–36), median viral load was 58,300 RNA copies/ml (IQR 3,100–185,500), and median CD4 T cell absolute count was 340 cells/mm^3^ (IQR 238–478). The cohort was predominantly female (79% female and 21% male). For studies of primary NK cell function, nine healthy HIV-1-negative individuals were recruited at Massachusetts General Hospital (Boston, MA) and at Brigham and Women’s Hospital (Boston, MA; IGTB cohort). HLA typing was performed by 454 sequencing, and KIR typing by real-time PCR.

### Sequencing of HIV-1 Gag Gene in Individuals Infected with HIV-1 Clade C

RNA was extracted from the blood plasma of patients infected with HIV-1 clade C, and the region p24 of the Gag gene was amplified by nested reverse transcription PCR as part of the *gag-protease* region of the virus. The entire PCR amplicon (including the p24 Gag region) was sequenced using BigDye Terminator Ready Reaction Mix V3 (Applied Biosystems) with primers described in detail elsewhere [30]. HIV-1 Gag sequences were obtained for 392 of the 406 studied individuals, and KIR genotyping was inconclusive for two of these patients. Our analysis is therefore based on the 390 study participants with full Gag sequencing and KIR/HLA genotyping. The underlying consensus clade C sequence was based on the HIV-1 consensus sequence of the 392 study participants. Gag-protease sequences obtained in this study are available in the GenBank database under nucleotide sequence accession numbers HM593106 to HM593510.

### Identification of KIR/HLA-Associated Sequence Polymorphisms within HIV-1 p24 Gag

A statistical approach that yielded a rank-ordered list of the most likely instances in which HLA/KIR combinations interacted to drive selection of specific viral amino acid residues was developed to identify combinations (1) in which specific HLA/KIR combinations were strongly associated with (or against) a particular viral amino acid and (2) where this association was stronger among individuals expressing both the HLA and KIR than among those expressing only the HLA or only the KIR. To this end, two separate tests were performed: (1) a phylogenetically corrected method to identify strong HLA+/KIR+ associations and (2) a logistic-regression-based interaction test to identify instances in which there was evidence of an interaction. The phylogenetically corrected method was run as previously described [31,32], using HLA/KIR, HLA, and KIR as independent variables and using forward selection with *p <* 0.05 as the addition criterion. The interaction test was performed using standard logistic regression, with significance determined by a likelihood ratio test for a model that included the interaction term versus one that did not. These two sets of tests were each independently applied to each viral amino acid observed at each site (see below for details of the binary encoding).

To combine these tests, we defined a new test statistic *t*_*i*_, which was simply the result of multiplying the *p*-values of the two tests for HLA-KIR-AA triple *i*. The significance (in terms of both *p*-values and false discovery rate) as estimated by *q*-values [33] was then estimated by comparing to null data, which were generated by repeating this approach on ten independent datasets. Each dataset was generated by randomizing the assignment of individuals to KIR haplotypes (thus preserving the HLA and KIR haplotype structures, as well as the strong HLA-escape-driven signals). The distribution of *q*-values included three associations with *q* ≤ 0.13, with the remainder with *q* > 0.35. As *q*-values are simultaneously conservative [34], and therefore allow post hoc selection of a threshold, we chose the top three associations to pursue further and report the *q*-values in Table 1. By combining these tests, we aimed to identify candidate polymorphisms selected only by the particular combination of HLA and KIR for further experimental analysis.

**Table 1:** KIR footprints in HIV-1 clade C sequence.

Footprint	Protein	Amino Acid Position	KIR/HLA Association	Consensus Amino Acid^a^	Variant Amino Acid^b^	*q*-Value
I	Gag	303	KIR2DL3/HLA-C*03:04	T^c^	V^d^	0.13
II	Gag	340	KIR2DL3/HLA-C*03:04	G	A	0.13

^a^HIV-1 consensus sequence in 392 study participants.

^b^Dominant variant.

^c^Consensus T was also significantly associated with the lack of encoding KIR2DL3/HLA-C*03:*04* (*q* = 0.1).

^d^Variant amino acid A at Gag_303_ was significantly associated to an HLA-C*03:*04* genotype alone (*p* < 0.001), but not in combination with KIR2DL3.

To reduce the number of tests, we applied a number of filters to the data as a predefined pre-processing step. Starting with KIR/HLA combinations for which the KIR molecule was known to bind to the HLA molecule [35], we further limited the tests to HLA-KIR-AA combinations that exhibited enough variation to yield meaningful tests and to reduce the computational burden. To this end, we arbitrarily chose ten as a minimum-count threshold for the HLA and KIR alone variables. We limited our tests to HLA-AA and KIR-AA combinations for which there were at least ten individuals in each of the following groups: HLA+, HLA−, KIR+, KIR−, AA+, AA−. For the combined HLA/KIR interaction test, we applied a stronger minimum count of six individuals in the following groups: HLA+/KIR+, AA+, AA−. Although arbitrary, these minimum-count thresholds were prespecified, and we did not explore other thresholds, so as to preserve the fidelity of the statistical analyses.

Amino acids were treated as binary variables to reduce the number of parameters required in the models, and separate models were created for all observed amino acids (subject to the minimum-count filter). Sites for which a mixture of amino acids was observed were treated as non-consensus. For example, for a site in which *X*, *Y*, and *Z* were amino acids that passed the minimum-count filter and in which *X* matched the cohort consensus, the mixture {*X*, *Y*} would yield the encoding [*X* = 0, *Y* = 1, *Z* = 0], while {*Y*, *Z*} would yield the encoding [*X* = 0, *Y* = 1, *Z* = 1]. Overall *n* = 13,826 KIR-HLA-AA triples were tested, with *n*_*r*_ = 182,734 tests in the randomized KIR runs (*n*_*r*_ ≠ 10*n* because of the HLA+/KIR+ ≥ 6 filter).

### Cell Lines and Primary Natural Killer Cell Populations Used

The HLA class I—deficient human B cell line 721.221 stably transduced with ICP47 was kindly provided by Emmanuel J. H. J. Wiertz (Department of Medical Microbiology, University Medical Center, Utrecht, The Netherlands). ICP47 is an early protein of HSV-1, which blocks loading of endogenous peptide onto HLA class I at the peptide binding site of TAP [36,37]. For the generation of a 721.221-ICP47 cell line stably expressing HLA-C*03:04, 293T cells (ATCC) were transfected with VSV-G, PCG (kindly provided by Thomas Pertel, Dana-Farber Cancer Institute), and pMIP, a retroviral transfer vector containing the HLA-C*03:*04* gene (kindly provided by Todd Suscovich, Ragon Institute of MGH, MIT and Harvard). Retroviral supernatant was harvested at day three after transfection and further used to transduce 721.221-ICP47 cells to obtain stable expression of HLA-C*03:04. Transduced cells were selected in 1.5 μg/μl puromycine and maintained in RPMI medium 1640 (Sigma-Aldrich) supplemented with 10% heat-inactivated fetal calf serum (Sigma-Aldrich), 2,500 U/ml penicillin, 2,500 μg/ml streptomycin, and 100 mM L-glutamine (Cellgro) at 37°C under 5% CO_2_.

### HLA-C*03:04 Stabilization and KIR2DL3-Fc Binding Assays

721.221-ICP47-C*03:04 cells were washed twice to remove any peptides remaining from FCS-supplemented medium and were subsequently pulsed with 100 μM of the indicated HIV-1 peptides in non-supplemented RPMI for 20 h at 26°C. Cells cultured with previously described HLA-C*03:04-stabilizing self-peptides (GAVDPLLAL [GAL] and GAVDPLLKL [GKL]) [9] were used as positive controls, while cells cultured with an influenza peptide (ILRGSVAHK) and in the absence of peptides were used as negative controls. After staining with the anti-pan-HLA antibody W6/32 (BioLegend), cells were fixed in 4% paraformaldehyde and analyzed by flow cytometry to quantify HLA class I expression. Titration experiments were performed using 100 μM, 10 μM, 1 μM, and 0.1 μM peptide, as indicated. KIR binding assays with the candidate peptides and controls were performed after 20 h of HLA class I stabilization. Cells were stained using 2.5 μg of KIR2DL3-Fc (recombinant human KIR2DL3-IgG-Fc chimera; R&D Systems, KIR2DL3*001 subtype) for 1 h on ice, followed by a secondary staining with anti-human-Fc antibody (PE-conjugated IgG goat anti-human polyclonal antibody, Invitrogen), as described previously [3]. Following fixation of cells in 4% (w/v in PBS) paraformaldehyde (Affymetrix), flow cytometric analysis was performed using a BD LSR II.

### Assessment of Primary Natural Killer Cell Activation

To determine activation of primary NK cells following exposure to target cells pulsed with different peptide variants, NK cell degranulation assays measuring CD107a expression were performed using freshly isolated and purified NK cells from nine healthy *KIR2DL3+* individuals [38]. Primary NK cells were isolated by incubating whole blood with RosetteSep Human NK Cell Enrichment Cocktail (Stemcell Technologies) for 20 min at room temperature, followed by Histopaque-1077 (Sigma) density gradient centrifugation. NK cells were rested overnight in RPMI medium 1640 (Sigma-Aldrich) supplemented with 10% heat-inactivated fetal calf serum (Sigma-Aldrich), 2,500 U/ml penicillin, 2,500 μg/ml streptomycin, 100 mM L-glutamine (Cellgro), and 1.0 ng/ml IL-15 (Cellgro). Subsequently, NK cells (1 × 10^5^) were co-incubated with peptide-pulsed 721.221-ICP47-C*03:04 cells (5 × 10^5^) at an effector:target ratio of 1:5 in RPMI containing anti-human CD107a-PE-Cy7 (12.5 μl/ml) and monensin (1.5 μl/ml, BD GolgiStop). Cells were incubated for 6 h at 26°C in 5% CO_2_. Cells were stained with 7AAD, anti-CD3-PB, anti-CD16-BV785, anti-CD56-BV605, anti-CD14/19-BV510, anti-KIR2DL2/3-PE, and anti-KIR2DL3-APC, washed, fixed with 4% (w/v in PBS) paraformaldehyde (Affymetrix), and analyzed by multiparameter flow cytometry using a BD LSR II.

### Analysis of Binding Affinities via Surface Plasmon Resonance

Surface plasmon resonance (SPR) measurements were made using a Biacore 3000 system; the experimental buffer used was HBS-EP (Biocore). To assess binding of the viral variants to KIR2DL3, biotinylated HLA-C*03:04 monomers (42 kDA) refolded with the peptides YVDRFFKTL, YVDRFFKAL, and YVDRFFKVL (Immudex) were immobilized onto a Streptavidin sensor chip (GE Healthcare) to approximately 500 response units for optimal responses. A blank flow cell with no immobilized ligand was used as a reference flow cell. Injections of 60 μl of KIR2DL3-Fc (recombinant human KIR2DL3-IgG- Fc chimera; R&D Systems, 102.4 kDA) diluted in PBS to a concentration of 10 μg/ml were performed at a flow rate of 20 μl/min, with a subsequent 10-min run of buffer to allow sufficient dissociation. Due to described instability after acid treatment of the HLA-C/peptide complex, acidic regeneration of the chip was not performed [39]. As a last step, the amount of each HLA monomer refolded with the respective epitope variant immobilized on the chip was determined by HC10 antibody staining. Raw sensograms were corrected by double referencing (subtracting from the reference flow cell response and from the PBS injection response). Given the dimeric nature of the KIR2DL3 analytes, a biphasic model was used to obtain the fit; binding constants are given as mean ± standard error of the mean (SEM). Experiments were performed at 25°C.

### Computational Modeling

All-atom, explicit solvent molecular dynamic simulations were used to obtain a detailed atomistic understanding of the structural and dynamic properties that modulate the specificity of binding of the three-way interaction between KIR2DL3, HLA-C*03:04, and peptide. An experimentally resolved crystal structure (Protein Data Bank ID 1EFX) [9] of KIR2DL2, in complex with its class I ligand HLA-C*03 and a self-peptide (GAL), was used as a structural template for all the studies herein and was adapted to fit KIR2DL3 modeling. A free energy perturbation (FEP) procedure was followed to obtain differences in the binding free energy (ΔΔG) between MHC with wild-type or mutant peptide (pMHC) and the KIR molecule. Further simulation details can be found in S1 Text, describing the modeling of the three-way interaction between KIR2DL3/HLA-C*03:04/peptide. S1 Fig shows the stability of the 1EFX structure (two KIR in complex with HLA-C*03 presenting GAVDPLLAL. S2 Fig illustrates the differences in the free energy of binding upon mutations of the self-peptide GAVDPLLAL. In S3 Fig we show the stability of the T_Gag303_ (YTL)–loaded HLA complex with KIR2DL3 binding. S4 Fig displays the insensitivity of the peptide binding groove to the identity of the peptide. S1 Text describes the details of the computational modeling.

### Data Acquisition and Analysis

Flow cytometry data were analyzed using FlowJo software version 10.0.6 (Tree Star), and statistical analysis was performed using GraphPad Prism 6 (GraphPad Software). HLA stabilization values are shown as mean relative fluorescence intensity; error bars represent standard deviation (SD). Relative fluorescence intensity was calculated by dividing the geometric mean fluorescence intensity (gMFI) of HLA expression in the presence of the tested peptide by the gMFI of stained target cells without peptide added in each respective assay. Binding of KIR2DL3-Fc is represented as percent of KIR2DL3+ cells ± SD. NK cell degranulation values are expressed as relative degranulation, by dividing the percentage of CD107a+ NK cells in a sample by the percentage of CD107a+ NK cells in the negative control sample from the same individual (where the non-binding GKL peptide was added). A repeated-measures one-way ANOVA was subsequently performed to compare the responses between the wild-type group and the groups of the two variant peptides, for the KIR2DL3+ and KIR2DL3− NK cells, respectively. This was followed by a Tukey’s post hoc test. Each individual was sampled one time.

## Results

### Identification of KIR/HLA Class I—Associated Sequence Polymorphisms within the HIV-1 Clade C p24 Gag Sequence

Previous studies have demonstrated that HIV-1 Gag represents an important target for cell-mediated immunity against HIV-1, including in individuals infected with HIV-1 clade C [40], resulting in viral escape from CD8+ T cell—mediated immune pressure by selection of sequence polymorphisms [27,30,41–46]. To determine the mechanism by which KIR+ NK cells might also impose immune pressure on HIV-1, we sequenced autologous p24 Gag genes from 392 individuals from Durban, South Africa, chronically infected with untreated HIV-1 clade C [30], for whom high-resolution HLA class I and KIR type information was available. Given the size of the cohort, we sought to identify sequence polymorphisms associated with the presence of specific KIR/HLA class I combinations in this population, rather than with the presence of the respective KIR allele or HLA class I allele alone [28], to guide further functional experiments. We therefore developed an exploratory statistical approach to identify sequence polymorphisms overrepresented in individuals with both the respective KIR and HLA class I alleles, but not in individuals with either the HLA class I allele alone or the KIR allele alone (or neither of them). This approach of combining the phylogenetically corrected method and the logistic-regression-based interaction test was chosen because, by themselves, the phylogenetically corrected method yielded a number of associations that were driven by the HLA allele alone, matching well-characterized T cell escape mutations, and the interaction test alone yielded no results that were significant after correcting for multiple tests (*q* = 1 for all tests).

The combined approach was subsequently applied to all 13,826 KIR/HLA polymorphism combinations in HIV-1 p24 Gag, and yielded three associations at two sites (Gag_303_ and Gag_340_) that were considered significant given the number of hypotheses tested (*q* ≤ 0.13; indicating a 13% false discovery rate) and were chosen because the next most significant association had a *q*-value of 0.35, providing a natural threshold. A combined genotype encoding *KIR2DL3* and HLA-C*03:*04* was associated with a valine at position Gag_303_ and an alanine at position Gag_340_, while not having the combined genotype was associated with having the wild-type threonine at position Gag_303_. A sequence change to having alanine at Gag_303_ was furthermore significantly related to having the HLA-C*03:*04* allele (*p* < 0.001), but upon combining with a KIR allele, significance was lost, as it was selected only in an HLA-C*03:04-related manner. In contrast, the sequence polymorphism encoding valine at Gag_303_ was solely significant for the combined KIR/HLA genotype, whereas no effect for this variant was observed among individuals possessing the HLA-C*03:*04* allele alone.

**Fig 1:**
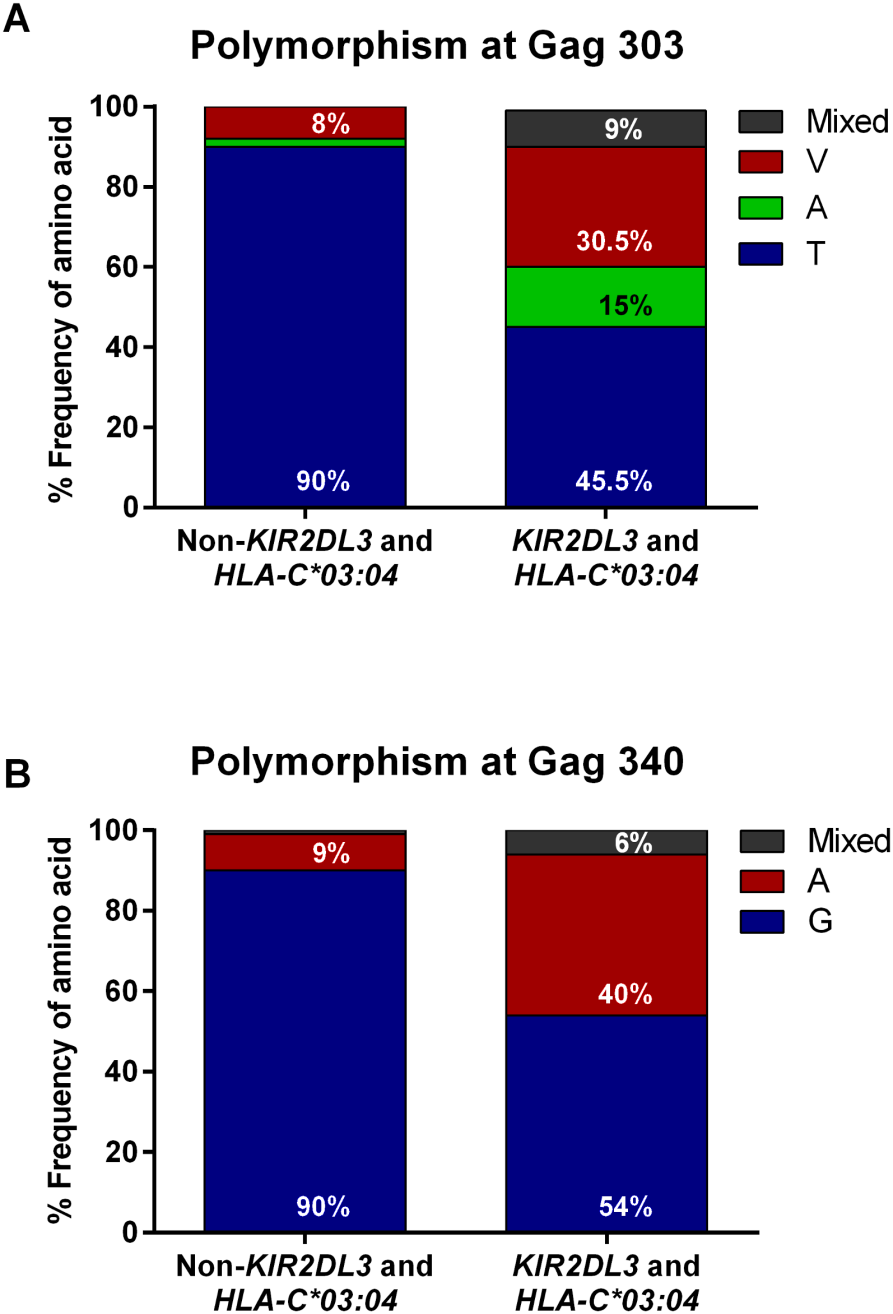
Distribution of frequencies of wild-type and variant Gag sequences in viruses from KIR2DL3+/HLA-C*03:*04+* individuals compared to individuals not possessing the combined KIR2DL3/HLA-C*03:*04* genotype. (A) A valine (V) at amino acid position 303 of HIV-1 Gag was significantly (*p <* 0.001, Fisher`s exact test) overrepresented in KIR2DL3+/HLA-C*03:*04+* individuals (*n* = 33) compared to individuals not possessing this combined genotype (non-*KIR2DL3* and HLA-C*03:*04*, *n* = 348), 90% of whom had virus with the consensus sequence wild-type threonine (T) in that position (*n* = 312). Furthermore, an alanine (A) variant at position Gag_303_ was present in five of the 33 individuals in the KIR2DL3+/HLA-C*03:*04+* group (15%). The percentage of individuals whose virus showed mixed residues at position Gag_303_ was 0% in KIR2DL3−/HLA-C*03:*04*− individuals and 9% in KIR2DL3+/HLA-C*03:*04+* individuals. (B) An alanine (A) at position 340 of HIV-1 Gag was significantly (*p <* 0.001, Fisher’s exact test) overrepresented in KIR2DL3+/HLA-C*03:*04+* individuals (*n* = 35) compared to individuals not possessing this combined genotype (non-*KIR2DL3* and HLA-C*03:*04*, *n* = 353), 90% of whom had virus with the consensus sequence wild-type glycine (G) in that position (*n* = 316). Two patients in the KIR2DL3−/HLA-C*03:*04*− subset had neither the wild-type nor the G_Gag340_A variant and are not shown in this graph. The percentage of individuals whose virus showed mixed residues at position Gag_340_ was 1% in KIR2DL3−/HLA-C*03:*04*− individuals and 6% in KIR2DL3+/HLA-C*03:*04+* individuals. Conclusive KIR2DL3 typing was not available for two out of the 392 study participants; these two participants were excluded from further analysis.

More than half of the viruses from individuals with the KIR2DL3/HLA-C*03:*04* genotype (18/33) had a sequence polymorphism in amino acid position 303 of p24 Gag, with a T_Gag303_V mutation in 39.5% of individuals (10/33) and a T_Gag303_A variant in 15% of individuals (5/33) (Fig 1A). Mixed variant and wild-type residues within the same patients were treated as escape in this position (3/33), as mixed residues suggested ongoing but not yet fully fixed escape. In contrast, 90% of individuals (312/348) not possessing this combined KIR/HLA genotype had virus with the T_Gag303_ wild-type sequence (*p <* 0.001, Fisher’s exact test; Fig 1A), and mixed residues were seen in virus from only two out of 348 patients. At amino acid position 340 of p24 Gag, 40% of the infected individuals with the KIR2DL3/HLA-C*03:*04* genotype had virus with a G_Gag340_A variant (14/35), compared to only 9% of KIR2DL3−/HLA-C*03:*04*− participants (33/353) (Fig 1B; *p <* 0.001, Fisher’s exact test), with the percentage of mixed residues at this position changing from 1% (4/353) to 6% (2/35) in the KIR2DL3/HLA-C*03:*04* genotype. Taken together, these statistical analyses using different models revealed polymorphisms within the HIV-1 clade C p24 Gag sequence associated with the presence of a specific combined *KIR/HLA* genotype in the studied population, but not with the presence of the respective KIR or HLA class I allele alone.

### The T_Gag303_ Variants Are Contained within an HLA-C*03:04-Presented Epitope and Are Equally Well Presented by HLA-C*03:04

To determine whether the two identified KIR/HLA-associated sequence polymorphisms within HIV-1 p24 Gag were contained within epitopes presented by the respective HLA class I molecules, we initially used HLA binding prediction models to identify potential epitopes. The amino acid polymorphism at position 303 of Gag was located within a previously described HLA-C*03:04-restricted CD8+ T cell epitope [47,48], and the wild-type epitope sequence YVDRFFKTL was predicted as a binder of HLA-C*03:04 using the NetMHCpan 2.8 predictor tool [49]. Notably, the sequence polymorphisms T_Gag303_V and T_Gag303_A were located at position P8 of the nonamer peptide, a position within the peptide for which the crystal structure of HLA-C*03:04 and KIR2DL2—an allele closely related to KIR2DL3—indicates an important role for KIR binding [9]. The epitope containing this footprint is highly conserved in HIV-1 clade C sequences published in the Los Alamos HIV Sequence Database (http://www.hiv.lanl.gov), with variation occurring almost exclusively at position P8 of the nonamer. Also, the second KIR2DL3/HLA-C*03:*04*-associated sequence polymorphism at amino acid position 340 of Gag was contained within a nonamer peptide (RALGPGATL) that showed a high binding affinity to HLA-C*03:04 using the NetMHCpan 2.8 restriction tool. As it had not been previously described as an HLA-C*03:04-restricted HIV-1 epitope, we tested 8- to 11-amino-acid-long peptides containing the G_Gag340_ wild-type or the G_Gag340_A variant sequence, and were able to confirm that the nonamer RALGPGATL was the optimal minimal epitope binding to HLA-C*03:04 at the lowest peptide concentration (S5A and S5B Fig). However, the sequence polymorphism at position 340 of Gag was located in position P6 of the HLA-C*03:04-presented epitope, a position not classically reported to be relevant for KIR binding to HLA-C*03:04.

**Fig 2:**
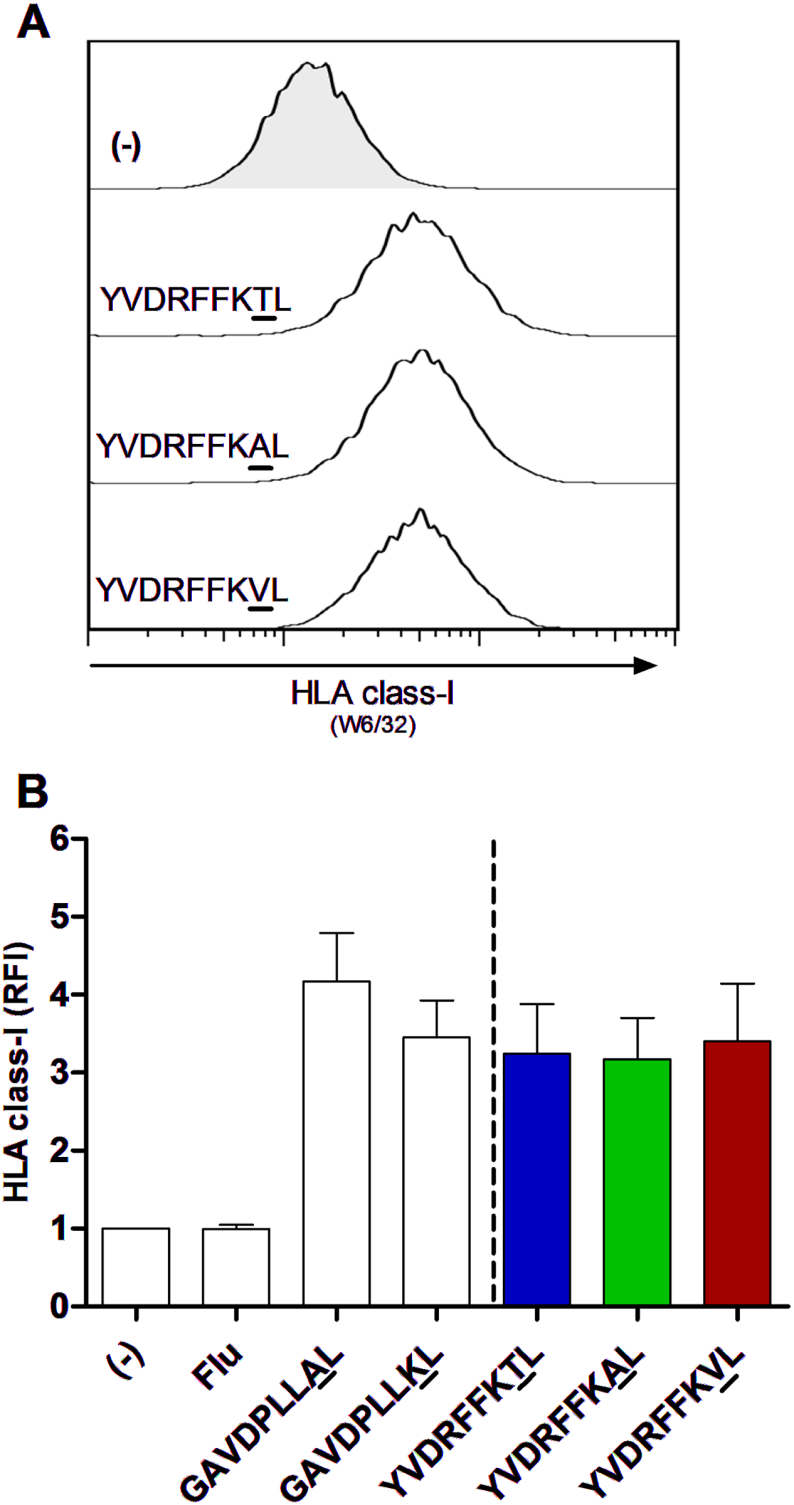
Equal HLA-C*03:04 stabilization on TAP-blocked 721.221-ICP47-C*03:04 cell line with HIV-1 p24 Gag T_Gag303_ wild-type and variant peptides. (A) Representative histograms of HLA-C*03:04 stabilization with T_Gag303_ wild-type and T_Gag303_A and T_Gag303_V variant peptides compared to addition of no peptide (−). HLA-C*03:04 surface levels were determined by flow cytometry using an anti-pan—HLA class I antibody (clone W6/32). Peptides were added at a saturating concentration of 100 μM. (B) Quantification of HLA-C*03:04 stabilization in the presence of T_Gag303_ wild-type and T_Gag303_A and T_Gag303_V variant peptides, as well as positive endogenous control peptides for HLA-C*03:04 stabilization (GAVDPLLAL and GAVDPLLKL) and a non-HLA-C*03:04-stabilizing influenza-derived control peptide (Flu, ILRGSVAHK). Data represent mean of five experiments with error bars indicating the SD. Relative fluorescence intensity (RFI) was calculated as the gMFI of the sample divided by the gMFI of 721.221-ICP47-C*03:04 cells stained in the absence of peptide.

We next investigated whether sequence variants in these epitopes binding to HLA-C*03:04 modulated the level of HLA expression on HLA-C*03:04+ cells. Using 721.221 cells, an HLA class I—devoid cell line, transduced with a single HLA class I gene (HLA-C*03:04) and ICP47 (an early HSV-1 protein known to block TAP [50]), allowed us to determine the ability of exogenously added peptides to bind to and stabilize HLA-C*03:04. As positive controls, the endogenous self-peptides GAVDPLLAL (GAL) and GAVDPLLKL (GKL), previously known to bind to HLA-C*03:04 [9], were used. 721.221-ICP47-C*03:04 cells were pulsed with the wild-type epitope YVDRFFKTL and its variants YVDRFFKVL and YVDRFFKAL and tested for HLA-C*03:04 stabilization by staining with the anti-pan—HLA class I antibody W6/32 (Fig 2). Pulsing with wild-type and variant peptides induced up to 4-fold higher expression of HLA-C*03:04 on the cell surface as compared to non-peptide-pulsed 721.221-ICP47-C*03:04 cells or 721.221-ICP47-C*03:04 cells loaded with the negative control peptide. More importantly, no differences in HLA surface expression were observed between the YVDRFFKTL, YVDRFFKVL, and YVDRFFKAL peptides, suggesting a stable and similar presentation of these three epitope variants by HLA-C*03:04 (T [mean 3.24 ± 0.64 SD] to A [mean 3.17 ± 0.53 SD], *p* = 0.996; T to V [mean 3.40 ± 0.74 SD], *p* = 0.681; A to V, *p* = 0.907). Titration experiments for HLA-C*03:04 stabilization using serial peptide dilutions confirmed equal HLA stabilization induced by wild-type and variant peptides containing the Gag_303_ polymorphism at non-saturating levels (T [mean 2.60 ± 1.45 SD] to A [mean 1.84 ± 0.79 SD], *p* = 0.732; T to V [mean 2.17 ± 1.36 SD], *p* = 0.229; A to V, *p* = 0.946; ANOVA/Tuckey’s post hoc test), indicating unaltered binding affinity despite the amino acid mutation in position P8 of the peptide. In contrast, RALGPGATL, the second HLA-C*03:04-restricted epitope containing position Gag_340_, bound as wild-type sequence with significantly higher affinity to HLA-C*03:04 than the epitope containing the sequence variant, RALGPAATL, when using non-saturating peptide amounts (G [mean 4.24 ± 0.46 SD] to A [mean 2.72 ± 0.81 SD], *p* = 0.006; paired, two-tailed *t*-test with 1 μM peptide; S5C Fig).

Taken together, these data demonstrate that the statistical approaches used identified two KIR2DL3/HLA-C*03:*04*-associated sequence polymorphisms within HIV-1 p24 Gag that were indeed contained within HLA-C*03:04-presented HIV-1 epitopes, providing experimental data that validate the statistical approach. While the KIR2DL3/HLA-C*03:*04*-associated T_Gag303_V and T_Gag303_A sequence polymorphisms had no impact on binding to and expression of HLA-C*03:04, the G_Gag340_A mutation reduced the ability of the epitope to bind to HLA-C*03:04. As the subsequent experimental approach used to assess the consequences of peptide variations on KIR binding relied on equal presentation of variant and wild-type peptides by HLA-C*03:04 for conclusive results, assessment of the consequences of the G_Gag340_A mutation for KIR2DL3 binding was not feasible. We therefore selected the T_Gag303_ variants to determine the consequences of the KIR2DL3/HLA-C*03:*04*-associated sequence polymorphisms for KIR/HLA interactions and NK cell function.

### The KIR2DL3/HLA-C*03:04-Associated T_Gag303_V Mutation Results in Enhanced Binding of the Inhibitory KIR2DL3 Receptor

**Fig 3:**
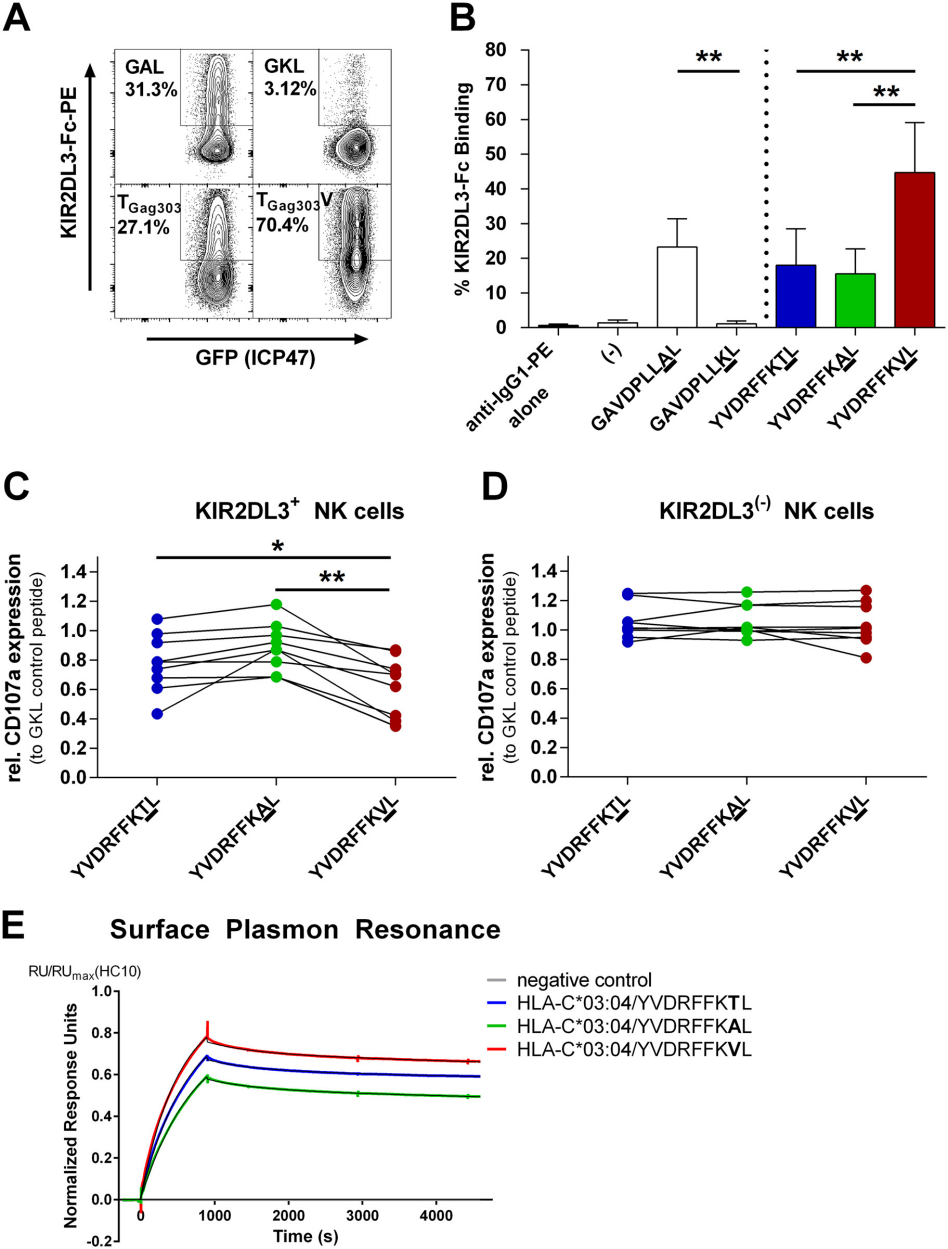
Binding of KIR2DL3-Fc and inhibition of primary KIR2DL3+ NK cells is significantly stronger when target cells are pulsed with the T_Gag303_V variant peptide. (A) Representative dot plots of KIR2DL3-Fc staining of 721.221-ICP47-C*03:04 cells pulsed with positive control peptide GAL, negative control peptide GKL, T_Gag303_ wild-type peptide, or T_Gag303_V variant peptide. Staining was measured by flow cytometry after addition of a secondary PE-conjugated anti-IgG antibody. All cells express GFP, indicating successful transduction of ICP47 and thus optimal blockade of TAP. (B) Quantification of KIR2DL3 binding expressed as percent of cells binding KIR2DL3-Fc. Means of six independent experiments, with error bars representing SD, are shown. Binding of KIR2DL3-Fc was significantly stronger to 721.221-ICP47-C*03:04 cells pulsed with the variant T_Gag303_V (YVDRFFKVL) peptide than to those pulsed with wild-type (YVDRFFKTL; *p* = 0.002) or T_Gag303_A (YVDRFFKAL; *p* = 0.002) peptide. (C and D) Degranulation of primary KIR2DL3+ (C) and KIR2DL3− (D) NK cells measured as CD107a expression after incubation with 721.221-ICP47-C*03:04 cells pulsed with T_Gag303_ wild-type and T_Gag303_A and T_Gag303_V variant peptides. The percentage of CD107a+ NK cells in response to the respective peptide is normalized to the percentage of CD107a+ NK cells for KIR2DL3− and KIR2DL3+ NK cell subsets after co-incubation with target cells pulsed with GKL (GAVDPLLKL) control peptide. 721.221-ICP47-C*03:04 target cells presenting YVDRFFKVL significantly inhibit degranulation of KIR2DL3+ NK cells compared to targets pulsed with YVDRFFKTL (*p* = 0.0121) or YVDRFFKAL (*p* = 0.0019). The different peptide variants had no detectable effect on CD107a expression in KIR2DL3− NK cells. All *p*-values stated are adjusted for multiplicity of testing. Primary NK cells from nine different healthy *KIR2DL3+* participants were tested. (E) Sensogram of KIR2DL3 dimeric analyte binding to biotinylated HLA-C*03:04/YVDRFFKTL, HLA-C*03:04/YVDRFFKAL, and HLA-C*03:04/YVDRFFKVL monomers on a Streptavidin chip; an empty well served as negative control. The sensogram data are normalized to the amount of respective HLA monomer immobilized on the chip by HC10 antibody. Each fit was obtained using double reference subtraction and is shown as a black line. Affinity of the KIR2DL3/HLA-C*03:04/YVDRFFKVL protein-protein interaction was highest. *p < 0.05; **p < 0.01.

To assess the consequences of the T_Gag303_V polymorphism on KIR2DL3 binding to HLA-C*03:04, we stained with a fusion chimera of KIR2DL3 attached to and dimerized by the Fc portion of hIgG1 (KIR2DL3-Fc) after labeling 721.221-ICP47-C*03.04 cells with the three different peptide variants (YVDRFFKTL, YVDRFFKVL, and YVDRFFKAL), and quantified KIR binding using a secondary PE-conjugated anti-human-IgG antibody. The positive control peptide GAVDPLLAL (GAL), known to allow for binding to KIR2DL2, which has binding patterns similar to those of KIR2DL3 [3], was used as an internal control for the engagement of KIR2DL3. The corresponding HLA-C*03:04-binding variant GAVDPLLKL (GKL) did not allow for KIR2DL3 binding, while still stabilizing HLA-C*03:04 at similar levels, and thus was used as a negative control. The results of repeated (*n* = 6) KIR2DL3-Fc binding assays are summarized in Fig 3A and 3B. While the wild-type peptide YVDRFFKTL and the variant peptide YVDRFFKAL allowed for a certain degree of KIR2DL3 binding, the YVDRFFKVL variant peptide preferentially selected in KIR2DL3+/HLA-C*03:*04*+ individuals resulted in significantly stronger binding of KIR2DL3-Fc (T [mean 18.01 ± 10.45 SD] and V [mean 44.67 ± 14.42 SD], *p* = 0.002; Fig 3A and 3B). Binding of KIR2DL3-Fc to HLA-C*03:04 in complex with the T_Gag303_V variant even exceeded binding to HLA-C*03:04 in complex with the positive control peptide GAL. The T_Gag303_A variant, previously reported to mediate escape from cytotoxic T lymphocyte (CTL) recognition [47], did not enhance binding to KIR2DL3-Fc compared to the wild-type sequence (T to A [mean 15.46 ± 7.24 SD], *p* = 0.811). This is in line with our findings showing no statistical association of this variant with the KIR2DL3+/HLA-C*03:*04+* combined genotype, but rather only with HLA-C*03:*04* alone, which might hint at CD8 T cell—related escape. Taken together, these data demonstrate that the T_Gag303_V polymorphism selected in KIR2DL3+/HLA-C*03:*04+* individuals led to a considerable increase in binding of the inhibitory KIR2DL3 receptor to HLA-C*03:04 as compared to the wild-type sequence.

### The KIR2DL3/HLA-C*03:04-Associated T_Gag303_V Mutation Inhibits KIR2DL3+ NK Cell Degranulation

We subsequently investigated whether the stronger KIR2DL3-Fc binding to HLA-C*03:04 induced by the T_Gag303_V variant peptide altered the effector function of KIR2DL3+ NK cells. Primary NK cells from healthy KIR/HLA-typed donors were co-incubated with 721.221-ICP47-C*03:04 cells pulsed with the peptide variants YVDRFFKTL, YVDRFFKVL, and YVDRFFKAL. Degranulation was measured by CD107a expression on NK cells, and KIR2DL3+ and KIR2DL3− NK cell subsets were distinguished using an antibody specific for KIR2DL3. When evaluating the function of KIR2DL3+ NK cells, no significant differences in CD107a expression after stabilization with the wild-type (YVDRFFKTL) or YVDRFFKAL peptide were observed (T [mean 0.78 ± 0.07 SEM] to A [mean 0.89 ± 0.05 SEM], *p* = 0.086), reflecting the results of the KIR2DL3-Fc binding assays (Fig 3B). However, co-incubation with 721.221-ICP47-C*03:04 cells pulsed with the YVDRFFKVL variant peptide led to a significant inhibition of degranulation of KIR2DL3+ NK cells compared to the wild-type peptide (T to V [mean 0.63 ± 0.07 SEM], *p* = 0.0121; Fig 3C) and to variant YVDRFFKAL (A to V, *p* = 0.002). In contrast, the different peptide variants had no effect upon the activation of KIR2DL3− NK cells (Fig 3D), as expected (T [mean 1.06 ± 0.04 SEM] to A [mean 1.06 ± 0.04 SEM], *p* = 0.933; T to V [mean 1.04 ± 0.05 SEM], *p* = 0.828; A to V, *p* = 0.603). Overall, results from KIR2DL3 binding to peptide-pulsed 721.221-ICP47-C*03:04 cells and degranulation of KIR2DL3+ NK cells in response to peptide-pulsed 721.221-ICP47-C*03:04 cells were closely correlated for the different peptides tested (*R* = 0.76, *p* = 0.02). Altogether, the sequence polymorphism T_Gag303_V in p24 Gag selected for in individuals infected with HIV-1 clade C who have the combined genotype KIR2DL3/HLA-C*03:*04* resulted in a blunting of primary KIR2DL3+ NK cell responses against HLA-C*03:04-expressing cell lines, while effector function of KIR2DL3− NK cells remained unaffected.

### Surface Plasmon Resonance Displays Enhanced Binding of HLA-C*03:04-Presented T_Gag303_V to KIR2DL3 Dimers

In order to further validate our findings, we performed SPR analysis to quantitatively assess the affinity between KIR2DL3 and the HLA-C*03:04-presented wild-type and variant epitopes. Variant T_Gag303_V (YVDRFFKVL) had the highest affinity to KIR2DL3 of the tested epitope variants upon presentation by HLA-C*03:04, with a *K*_D_ = 77 ± 3 nM. This was followed by the wild-type peptide T_Gag303_ (YVDRFFKTL), with a *K*_D_ = 81 ± 3 nM. HLA-C*03:04 refolded with variant T_Gag303_A (YVDRFFKAL) exhibited the lowest binding affinity of the three peptides, with a *K*_D_ = 93 ± 4 nM. The kinetic data indicated that the higher affinity of T_Gag303_V to KIR2DL3 was driven by both faster complex formation and a lower dissociation rate. Together, these data indicate that the selected T_Gag303_V variant binds with higher affinity to inhibitory KIR2DL3 than the wild-type or variant T_Gag303_A epitope.

### Computational Modeling Results Can Predict Binding of Variant Peptides to KIR2DL3-Fc and Functional Outcome of NK Cell Activation

**Fig 4:**
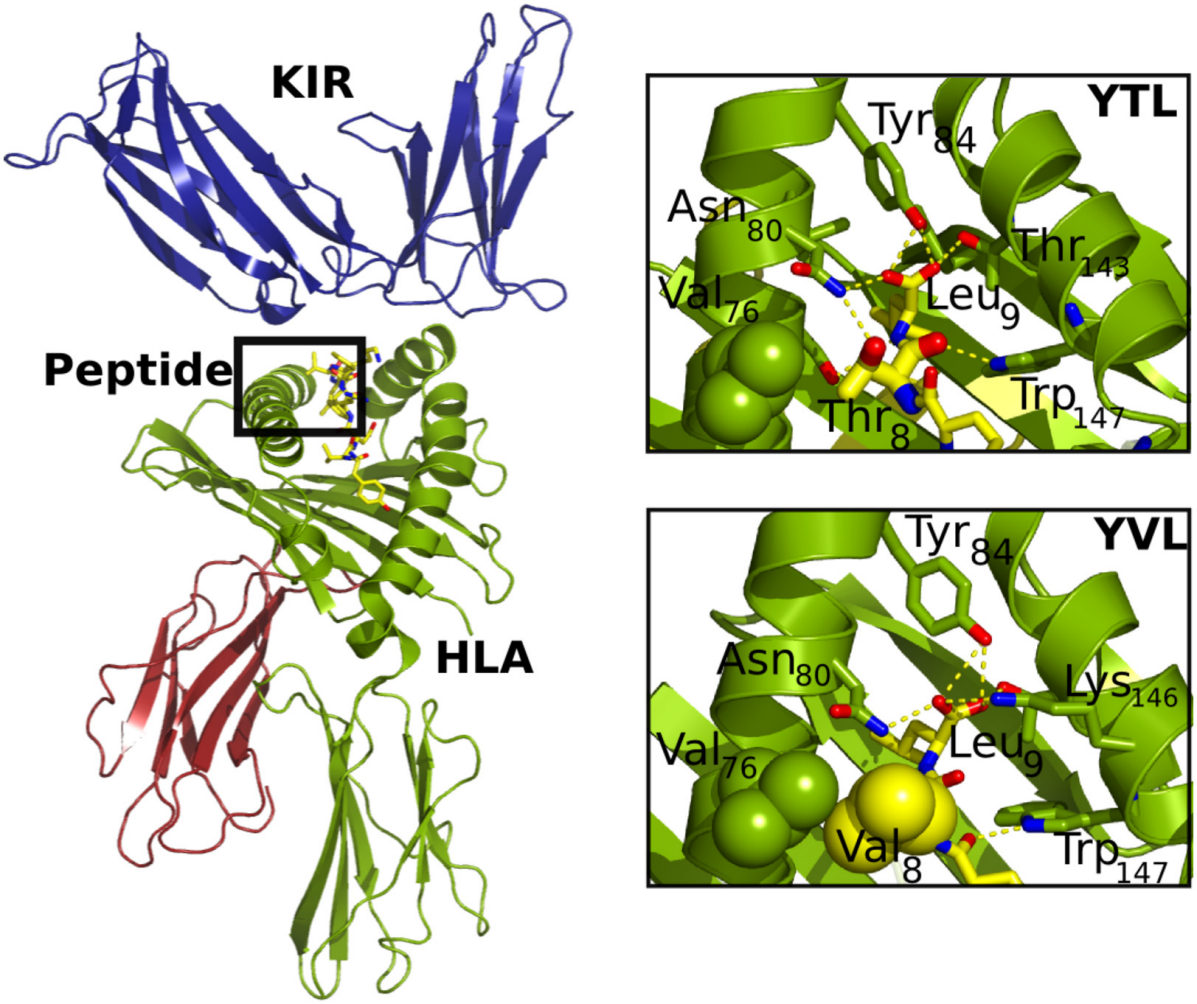
Structural details of the HLA/peptide/KIR three-way complex. (A) Overall structure of the HLA/peptide/KIR complex. The peptide is buried in the HLA class I binding grove, while KIR interacts with both helices of HLA class I as well as the C-terminus of the peptide. (B) Comparison of the interactions around the mutated viral residue (top: in fat sticks; bottom: in spheres). The YVDRFFKTL (YTL) wild-type peptide includes a hydrogen bond between the side-chain oxygens of Thr_8_ and Asn_80_, while the YVDRFFKVL (YVL) variant improves the hydrophobic packing against Val_76_. Interestingly, Asn_80_ participates in a hydrogen bond expected to confer allotype specificity to KIR2Ds.

The crystal structure of KIR2DL2 in conjunction with HLA-C*03:04 presenting a self-peptide (GAVDPLLAL) has been described previously [9]. Based on this structure, we used all-atom, explicit solvent molecular dynamic simulations adapted to the closely related KIR2DL3 structure, which differs by only 24 amino acid positions from KIR2DL2 (Immuno Polymorphism Database; https://www.ebi.ac.uk/ipd/kir/). We did this to obtain a detailed atomistic understanding of the structural and dynamic properties that modulate the specificity of binding between KIR2DL3 and HLA-C*03:04, with a particular interest in the dependence of the sequence of the HLA class I—presented peptide. First, to assess the validity and reach of the simulation protocol, two independent simulations were run for 50 ns each of the 1EFX structure containing an HLA-C*03:04 molecule loaded with GAL bound to a KIR2DL3 molecule. While some small inter-domain, hinge-like motions were observed in both simulations, each domain remained stable, as shown by Cα root mean square deviations (RMSDs) smaller than 0.15 nm (S1 Fig). Moreover, the binding surface between peptide-loaded HLA class I and KIR was unmodified during the length of the simulations, further supporting the thermal stability of the complex. KIR2DL3 binds orthogonally to one end of the peptide-binding groove, interacting with both the α1 and α2 helices of the HLA molecule and the C-terminus of the peptide (only residues 7 and 8), as shown in Fig 4. The complex is held together by strong charge complementarity between the KIR and corresponding HLA-C*03:04 surface, forming a dense network of salt bridges. Eight hydrogen bonds and four salt bridges formed between the negatively charged KIR surface and the positively charged HLA surface, and led to the strong recognition.

Once the binding mode was identified as adequate and stable under the simulation parameters employed, a rigorous FEP strategy was used to calculate the free energy changes of binding between the positive control peptide (GAVDPLLAL) and the negative control (GAVDPLLKL), along with three other peptide variants previously characterized experimentally for KIR2DL2 binding (GAVDPLLYL, GAVDPLLVL, and GAVDPLLSL) [9]. The results of the FEP calculations (S2 Fig) agree remarkably well with experimental results described by Boyington et al. [9] (S2 Fig), allowing for validation of both the current simulation parameters and the use of the FEP protocol to obtain the changes in binding free energy of the complex upon sequence variations of the self-peptide. Next, the endogenous self-peptide in the complex was replaced by the wild-type HIV-1 peptide YVDRFFKTL (T_Gag303_); details of the two-step preparation procedure, equilibration, and stability assessments are provided in S3 and S4 Figs and S1 Text. The overall shape of the HLA-C*03:04 binding pocket for the peptide remained highly conserved upon substitution of the peptide chain (S4A Fig), consistent with the experimental data from the HLA-C*03:04 stabilization assays (Fig 2). The peptide backbone, however, differed only in the position P5, which twisted significantly and became more exposed to the solvent (S4B Fig). Both termini of the peptide remained completely buried in the binding pocket, with the charged C-terminus of the peptide engaging in multiple hydrogen bonds with Tyr_84_, Tyr_123_, and Thr_143_ of HLA-C*03:04. The stabilized structure was then used to compute the changes in free energy (ΔΔG) upon binding of the wild-type sequence (T_Gag303_) and five different variants: the in-vivo-selected HIV-1 sequence polymorphisms (T_Gag303_V and T_Gag303_A), a naturally occurring minor variant T_Gag303_C (1.9%, Los Alamos HIV Sequence Database), and two additional experimental variants, T_Gag303_F and T_Gag303_G.

Compared to the wild-type sequence YVDRFFKTL, the T_Gag303_V mutant showed a moderate enhancement in binding affinity (ΔΔG = −0.51 ± 0.41 kcal/mol; Table 2), which agreed well with the experimental observations (Fig 3B and 3C; Table 2). The free energy decomposition revealed that the stability of binding of this mutation was mainly due to a favorable electrostatic term, which contributed about −1.31 kcal/mol. It turned out that this contribution was largely from the much less favored free state because of the loss of hydrogen bonds with the solvent in the free state. The bound state also lost some hydrogen bonds with water, but because of the compensation from the KIR, the overall loss in total hydrogen bonds was less significant. In contrast, the T_Gag303_A mutant slightly destabilized the complex (ΔΔG = 0.38 ± 0.34 kcal/mol), again in line with the experimental data. While threonine and valine are similar in size, the alanine residue is significantly smaller and induced a penalty in the overall favorable hydrophobic packing of the system. A similar situation occurred with the T_Gag303_G mutant (1.10 ± 0.74 kcal/mol), with an energetic profile comparable to that of the alanine variant. The bulky aromatic group introduced by the T_Gag303_F mutant resulted in a large entropic penalty due to less flexible Phe side chains. The mutations T_Gag303_A, T_Gag303_C, T_Gag303_F, and T_Gag303_G thus either decreased or completely destroyed the binding between HLA-C*03:04 and KIR2DL3 in our computational model, while the T_Gag303_V mutant selected in KIR2DL3+/HLA-C*03:*04+* individuals enhanced the binding to the inhibitory KIR2DL3 receptor in this model, in line with the experimental data.

**Table 2:** Experimental validation of modeling results.

Peptide	Mutation	KIR2DL3-Fc Binding^a^ (Mean ± SD)	KIR2DL3+ NK Cell Activity^b^ ^,c^ (Mean ± SEM)	ΔΔG (kcal/mol)			
				ΔΔG_total_ (Mean ± SEM)	ΔΔG_elec_	ΔΔG_vdW_	ΔΔG_coup_
**Self**	GKL	0.06 ± 0.03	1.70 ± 0.3	6.46 ± 1.13	3.31	3.26	−0.11
**Viral**	T_Gag303_V	2.74 ± 0.66	0.76 ± 0.08	−0.51 ± 0.41	−1.31	1.04	−0.22
	T_Gag303_A	0.90 ± 0.17	1.36 ± 0.33	0.38 ± 0.34	−0.24	0.79	−0.16
	T_Gag303_C	0.08 ± 0.06	1.45 ± 0.05	0.18 ± 0.52	−0.08	0.35	−0.09
**Modeling leads**	T_Gag303_F	0.23 ± 0.18	1.32 ± 0.19	3.52 ± 2.19	0.71	2.67	0.13
	T_Gag303_G	0.15 ± 0.12	1.27 ± 0.04	1.10 ± 0.74	0.23	0.96	−0.09

^a^Ratio of percent KIR2DL3-Fc binding of variant peptides to percent KIR2DL3-Fc binding of wild-type peptide T_Gag303_.

^b^Ratio of percent CD107a+ cells in group of KIR2DL3+ NK cells incubated with variant peptides compared to percent CD107a+ cells in group of KIR2DL3+ NK cells incubated with wild-type peptide.

ΔΔG_coup_, coupling free energy; ΔΔG_elec_, electrostatic free energy; ΔΔG_vdW_, van der Waals free energy.

Based on the KIR2DL3 binding predictions for the additionally tested minor variant T_Gag303_C, and the two experimentally designed peptide variants (T_Gag303_F and T_Gag303_G), we performed subsequent experimental validations using these peptides. As shown in Table 2, a close correlation between the experimental data and modeling data for the variants T_Gag303_F and T_Gag303_G was observed, similarly to the previously tested T_Gag303_ wild-type, T_Gag303_V, and T_Gag303_A peptides. Only the T_Gag303_C variant gave discordant results—with the experimental data showing a more drastic decrease in KIR2DL3-Fc binding compared to wild-type (mean 0.08 ± 0.06 SD) than predicted by the change in ΔΔG (0.18 ± 0.52 kcal/mol) (Table 2). The ΔΔG values resulting from the modeling studies and the KIR2DL3-Fc binding data from the experimental studies were correlated (*R* = 0.5), but this correlation did not reach significance given the discordant results for the T_Gag303_C variant. Excluding this variant resulted in a significant correlation between modeling results and experimental data (*R* = 0.8, *p* = 0.04). Taken together, the modeling data based on the KIR2DL2 crystal structure adapted to KIR2DL3 agreed very well with the observed experimental data, and also allowed for predictions about how peptide variants might modulate KIR/HLA class I interactions that could be experimentally validated.

## Discussion

Persistent HIV-1 infection is characterized by a continuously evolving struggle between immune recognition of the virus and viral escape from immune control. HIV-1 evades host immune pressure by numerous means, such as differential down-regulation of HLA class I by accessory gene products [51], adaptation to the cellular antigen-processing machinery [52], and selection of single amino acid sequence variants enabling evasion from T cell responses [44] or antibodies [53]. We recently demonstrated that HIV-1 can also adapt to KIR-mediated immune pressure [28], but the mechanisms by which viral sequence variations can reduce the ability of KIR+ NK cells to recognize HIV-1-infected cells remain unknown. We sought to further elucidate these mechanisms through the identification of viral sequence polymorphisms selected at the population level in infected individuals expressing specific combinations of KIRs and their HLA class I ligands, and identified two amino acid mutations in the HIV-1 clade C p24 Gag sequence that are significantly associated with the presence of distinct KIR/HLA combined genotypes. Functional studies demonstrated that the selection of the T_Gag303_V sequence polymorphism resulted in significantly better binding of the inhibitory NK cell receptor KIR2DL3 to HLA-C*03:04, and in inhibition of KIR2DL3+ primary NK cells. These functional data were supported by structural modeling of the KIR/HLA interaction in the context of wild-type and variant epitope sequences and validated by SPR analysis. Taken together, these results elucidate a novel mechanism by which HIV-1 can evade NK-cell-mediated immune pressure through the selection of single amino acid variants within HLA class I—presented epitopes that inhibit the function of NK cells expressing the respective KIR.

Previous studies have successfully employed statistical approaches to identify viral sequence polymorphisms associated with the expression of individual HLA class I molecules on a population level resulting from escape from CD8+ T cell—mediated immune pressure. These mutations can lead either to impaired presentation of viral epitopes by HLA class I molecules or abrogation of T cell receptor binding. Similar approaches have been used to identify viral adaptation to other host factors, including host restriction factors [54], and more recently also to KIRs that are expressed on NK cells and modulate NK cell function [28]. However, due to limitations in sample sizes, it was not possible to date to identify viral sequence polymorphisms associated with the presence of combined host factors, such as the highly polymorphic genes encoding HLA class I and KIR. KIRs bind to HLA class I molecules, with significant consequences for NK cell function, and HLA class I and KIR have co-evolved in humans, modulating the outcome of pregnancy, autoimmune diseases, and infectious diseases including HIV-1 [55,56]. As the interaction between KIR and HLA class I can be modulated by the HLA class I—presented peptides [3–8], we sought to investigate whether HIV-1 might exploit these peptide-dependent interactions through the selection of sequence polymorphisms that evade NK-cell-mediated immune recognition. We were confronted with several challenges: (1) the number of HLA/KIR/polymorphism combinations is large, yielding a large number of tests and therefore requiring large sample and effect sizes to reach statistical significance; (2) polymorphisms within HLA class I—presented viral epitopes that affect KIR binding may also affect CD8+ T cell recognition [57], thereby diluting the KIR-specific signal; and (3) the distribution of KIR and HLA is unbalanced. We addressed these challenges by first limiting the analysis to combinations where the KIR/HLA receptor/ligand pairs were already known. Second, we identified associations that were significantly more represented in participants with a combined KIR/HLA genotype than in the pooled group of participants possessing only one gene allele or being negative for both alleles. At the same time, we allowed for an only marginally greater representation of the variant in the combined genotype compared to an HLA+ background alone. Thereby, we established a valid approach excluding escape mutations driven solely by CD8+ T cell—mediated immune pressure while at the same time being sensitive enough to identify those sequence polymorphisms selected in a KIR/HLA interaction context. Using this approach, we were able to identify two KIR/HLA-associated sequence polymorphisms within the HIV-1 clade C p24 Gag sequence in a cohort of 392 treatment-naïve, chronically infected individuals from South Africa. Given the still very large number of KIR/HLA/polymorphism combinations that had to be tested (*n* = 13,826), we most certainly underestimated the total number of KIR/HLA-associated sequence polymorphisms within this cohort of 392 individuals. However, the identification of two sequence polymorphisms that were significantly associated with the presence of combined *KIR/HLA* genotypes provided us with the unique opportunity to study the functional consequences of these sequence variations for NK-cell-mediated immune recognition.

We first sought to determine whether the peptides holding the KIR2DL3/HLA-C*03:*04*-associated sequence variants were indeed presented by HLA-C*03:04. The Gag_303_ polymorphism was located within a described optimal HIV-1 CTL epitope restricted by HLA-C*03:04 [47]. Contrary to evasion from epitope-specific CD8+ T cells, published crystal structures of KIR2DL2 and HLA-C*03:04 showed that only the residues at the C-terminal end of the epitope were involved in KIR/HLA interactions [9]. In line with this, the amino acid substitution at Gag_303_ was the only major variant in the YVDRFFKTL epitope observed in published HIV-1 sequences, and, notably, the sequence polymorphism was located at the C-terminal end of the peptide at amino acid position 8 (P8). Furthermore, the selected sequence polymorphisms T_Gag303_V and T_Gag303_A did not change the ability of the respective epitopes to bind to HLA-C*03:04. In contrast, the second KIR2DL3/HLA-C*03:*04*-associated sequence mutation in RALGPGATL occurred at position P6 of the peptide, where it is less likely to affect KIR2DL3 binding, and surface stabilization of HLA-C*03:04 by the G_Gag340_A variant peptide was significantly reduced compared to that of the wild-type peptide, rendering it unfeasible to assess the consequences for KIR binding. Overall, these data suggested that, in contrast to the Gag_303_ polymorphisms, the Gag_340_ polymorphisms might not directly modulate KIR binding to HLA class I as a main mechanism of immune escape. Alternatively, the mutation at Gag_340_ might be a mutation disrupting or altering processing of a flanking epitope, as was described before for other HIV-1 sequence polymorphisms [58–60]. It is possible that this flanking epitope can be presented on HLA-C*03:04 and engage KIR2DL3, which would explain the association detected in the cohort. Furthermore, our experimental approach would not have detected alterations in HLA class I or KIR binding of the peptide resulting from differential posttranslational modifications of wild-type or variant peptides [61]. Taken together, the statistical approach used here allowed us to identify two KIR2DL3/HLA-C*03:*04*-associated sequence polymorphisms within HIV-1 p24 Gag, one of which (at Gag_303_) had no impact on epitope binding to HLA class I but was located in a position that modulated KIR2DL3 binding.

The Gag_303_ sequence polymorphisms had been previously described to be located in an epitope representing an immunodominant target for HIV-1-specific CD8+ T cells [29,48], and to be associated with evasion from CD8+ T cell—mediated immune pressure [47]. These studies showed that the T_Gag303_A polymorphism resulted in a reduction of recognition by epitope-specific CD8+ T cells, as well as a reduction in viral fitness [47], and in our dataset the T_Gag303_A variant was also strongly associated with the presence of HLA-C*03:04 alone (*p* = 0.0005) (but not in compound with KIR2DL3). In contrast, the T_Gag303_V variant that was selected in individuals with the KIR2DL3/HLA-C*03:*04* combined genotype had no impact on viral replication capacity but, surprisingly, restored recognition by HIV-1-specific CD8+ T cells to a level similar to that of the T_Gag303_ wild-type sequence in studies by Honeyborne et al. [47], raising the question of why this variant was selected in individuals with HLA-C*03:*04*, and ultimately represented the major variant in these individuals. Here we demonstrated that the occurrence of the T_Gag303_V mutation was not associated with the presence of the HLA-C*03:*04* genotype alone, but significantly enriched only in individuals with the combined KIR2DL3/HLA-C*03:*04* genotype. We furthermore demonstrated in functional studies that the T_Gag303_V variant significantly enhanced the binding of KIR2DL3 to HLA-C*03:04 molecules presenting the T_Gag303_V epitope and inhibited the function of KIR2DL3+ primary NK cells, strongly suggesting that KIR2DL3+ NK cells might have a role in driving viral evolution in this epitope. Interestingly, the mutation from threonine to valine can be achieved only by a two-step nucleotide mutation, necessarily passing through an intermediate variant such as alanine. This provides an explanation for why the T_Gag303_A variant was observed in a subset of the studied KIR2DL3+/HLA-C*03:*04+* population (Fig 1A, 15%), although it did not alter KIR2DL3 binding or KIR2DL3+ NK cell function compared to the wild-type sequence. Furthermore, coexistence of viral sequences containing T_Gag303_ wild-type and T_Gag303_A/V variants was observed by phylogenetic analysis in two of the KIR2DL3+/HLA-C*03:*04+* study participants, suggesting that T_Gag303_A represents a transitory stage that is subsequently replaced in the majority of KIR2DL3+/HLA-C*03:*04+* individuals by T_Gag303_V [47]. Taken together, these data reflect the critical interplay between CD8+ T cell—mediated and NK-cell-mediated immune pressure in the selection of sequence variations in HIV-1.

A limitation of our study is that no conclusions can be drawn as to when during natural infection the observed viral amino acid sequence changes were selected, as the time point of infection in the HIV-1-infected participants enrolled in this study was not known. Longitudinal studies will be required to address this question, and also to determine the consequences of these sequence mutations for viral fitness and in vivo control of HIV-1 replication. Future studies will also be needed to assess processing of the epitope variants and the kinetics of antigen presentation of the investigated HIV-1 sequence polymorphisms in natural infection, as well as the abundance of HLA class I presentation of these peptides on HIV-1-infected cells.

Using SPR analysis, we observed enhanced binding of T_Gag303_V to KIR2DL3 compared to the wild-type epitope. In line with our biological data, T_Gag303_A exhibited the lowest predicted monomeric affinity to KIR2DL3. Of note, recombinant KIR2DL3-Fc was used as a dimeric analyte in our assay, which increases the avidity to all HLA-C*03:04/peptide refolded monomers. Thus, we were only able to obtain monomeric affinity values using model-based calculations, which have inherent assumptions that can cause discrepancies relative to true monomeric affinities. This might in part explain the observed small differences in *K*_D_ values between the two key peptides. Based on the results from functional studies and SPR analysis, we subsequently employed a computational modeling approach to further understand the underlying thermodynamic changes caused by single amino acid substitutions in HLA-C*03:04-presented epitopes leading to altered KIR2DL3 binding on a molecular level. Modeling approaches showed that the mutation in position P8 of the YVDRFFKTL epitope did not notably alter the overall shape of the HLA-C*03:04 binding pocket, which can explain the equal stabilization of HLA-C*03:04 by the variant and wild-type peptides observed experimentally. Despite some controversy in the literature about the meaningfulness of breaking down the total free energy into electrostatic and van der Waals components, and the ambiguity associated with a path-dependent decomposition [62–64], we believe that breaking up the total binding free energy into various components allowed us to gain a more detailed understanding of the physical interactions involved in the peptide/HLA/KIR binding and to optimize our ability to predict the effects that these amino acid changes have on HLA class I presentation and KIR binding. The stronger affinity of HLA-C*03:04 in complex with peptides containing the T_Gag303_V variant to KIR2DL3 can thus largely be explained by unfavorable electrostatic interactions of the HLA class I molecule in the unbound state due to the hydrophobicity of valine compared to the wild-type threonine. Although a few hydrogen bonds between peptide and solvent were still lost upon engagement of KIR, this was compensated for by binding of KIR2DL3 to the HLA-C*03:04 molecule itself. Interestingly, one of the residues in the HLA class I molecule participating in these compensatory hydrogen bonds with KIR2DL3 was HLA-Asn_80_, which is the residue conferring allotype specificity of the HLA-C group 2 via binding to KIR Lys_88_. The strong long-range interactions of KIR2DL3 with the HLA-C*03:04 molecule itself, together with a few direct contacts between amino acids in the peptide (mainly P8, and to some extent P7) and KIR2DL3, demonstrate a completely different molecular mechanism from CD8+ T cell recognition, where the HLA class I—presented peptide has significantly more direct exposure to the T cell receptor [65]. While for most of the epitope variants a close correlation between the modeling data and the experimental KIR2DL3 binding data was observed (*R* = 0.8, *p* = 0.04), the modeling results for the T_Gag303_C variant did not fully agree with the experimental KIR2DL3-Fc binding data. Even though FEP analysis may have difficulties in catching small changes in binding affinity (~*k*_B_ *T* = 0.6 kcal/mol), such as in this particular case—which is likely due to force field accuracy and sampling issues—the overall trend predicted agreed well with the experimental data and provided novel insights into the structural and mechanistic changes on a molecular level occurring with distinct substitutions in the peptide sequence, and their consequences for KIR/HLA interactions and NK cell function.

Taken together, the results of this study in a large cohort of individuals from South Africa infected with HIV-1 clade C provide novel insights into the mechanisms by which HIV-1 can escape NK-cell-mediated immune pressure through the selection of sequence variants that enhance the binding of an inhibitory KIR to HLA class I/peptide complexes. This immune evasion mechanism might also be used by other pathogens with high mutation rates and potentially also in tumor immune evasion, as it demonstrates how specific mutations in an antigen can lead to the inhibition of KIR+ NK cell function. A better understanding of these molecular mechanisms will provide the rationale to use NK cells therapeutically, for example, in combination with KIR-blocking antibodies, which have already been tested in the oncology field in phase I clinical trials [66–68]. The computational models used here will furthermore allow the extension of these observations to other KIR/HLA interactions, facilitating the search for further virus- or tumor-derived peptides modulating NK cell function in an efficient and comprehensive screening system. Better understanding of the mechanisms that regulate the recognition of virus-infected or tumor cells by NK cells will facilitate approaches to harness the antiviral activity of NK cells in a manner more specific than previously thought possible.

## Supporting Information

S1 Fig Stability of the 1EFX structure (two KIRs in complex with HLA-C*03 presenting GAVDPLLAL). The simulations show a stable 3-D structure during the simulation time, with most of the deformations contained in hinge-like motions. (A) Overall Cα RMSD in the crystal structure versus time for two independent simulations. (B) Cα RMSD in the crystal structure on a per-domain basis. (C) Color labeling for the different domains considered.
(TIF)

S2 Fig Differences in the free energy of binding for mutations of the self-peptide GAVDPLLAL. Mutation of the p8 alanine to lysine or tyrosine results in a severe loss of binding affinity (in excess of 6 kcal/mol), with a large component of entropic contribution. Mutation to valine or serine results in more modest destabilization of the complex. (A) Binding free energy changes of each mutant with respect to GAL. (B) Decomposition of the free energy difference in the van der Waals and electrostatic contributions. The limited space around position 8 of the peptide induces preference for smaller peptides, reducing binding affinity for larger or bulky amino acids.
(TIF)

S3 Fig Stability of the T_Gag303_ (YTL)–loaded complex. The self-peptide in 1EFX was replaced with the T_Gag303_V (YVL) mutant in a two-step process (see Methods) and simulated for a total of 100 ns. Here, results for a 30-ns trajectory are shown. The black curves show the overall deformation, and the other colors follow the scheme described in S1 Fig. Upon replacement and equilibration, the system remained stable at the interface, displaying small variations mainly contained in the α3 domain of the HLA molecule.
(TIF)

S4 Fig (A)The peptide binding groove is largely insensitive to the identity of the peptide. A superposition of the self-peptide (GAL), the viral wild-type sequence (YTL), and a selected mutant (YVL) is shown. (B)The peptide recognition is provided by hydrogen bonds in the two termini (not shown) but allows for large variability in the central region of the peptide (residues P4, P5, and P6).
(TIF)

S5 Fig Identification of optimal epitope containing G_Gag340_ and levels of HLA-C*03:04 presentation. The optimal epitope was determined by the level of HLA-C*03:04 stabilization on TAP-blocked 721.221-ICP47-C*03:04 target cells pulsed with peptides of differing length containing wild-type amino acid G (A) or variant amino acid A (B) at position Gag_340_. The HLA stabilization assay was performed with decreasing concentrations until non-saturating levels of peptide labeling were reached. We identified RALGPGATL and RALGPAATL as the optimal HLA-C*03:04-restricted epitopes. (C) The wild-type peptide RALGPGATL stabilized HLA-C*03:04 expression on 721.221-ICP47-C*03:04 cells significantly better than the variant epitope RALGPAATL at non-saturating concentrations of 1 μm (G [mean 4.24 ± 0.46 SD] to A [mean 2.72 ± 0.81 SD), *p* = 0.006) and 0.1 μM (G [mean 2.26 ± 0.39 SD] to A [mean 1.29 ± 0.19 SD], *p* = 0.008) as measured by paired, two-tailed *t*-test. HLA-C*03:04 surface expression was determined flow cytometrically by staining with the anti-pan-HLA antibody W6/32 (*n* = 3).
(TIF)

S1 Text Details of the computational modeling. (PDF)

## Abbreviations

CTL: cytotoxic T lymphocyte
FEP: free energy perturbation
gMFI: geometric mean fluorescence intensity
HLA: human leukocyte antigen
IQR: interquartile range
KIR: killer-cell immunoglobulin-like receptor
NK: natural killer
RMSD: root mean square deviation
SD: standard deviation
SEM: standard error of the mean
SPR: surface plasmon resonance
