# Supplementary figures and images for: Selection of an HLA-C*03:04-Restricted HIV-1 p24 Gag Sequence Variant Is Associated with Viral Escape from KIR2DL3+ Natural Killer Cells: Data from an Observational Cohort in South Africa
Source: PLoS Med. 2015 Nov 17;12(11):e1001900. doi: 10.1371/journal.pmed.1001900 (PMC4648589; doi:10.1371/journal.pmed.1001900)

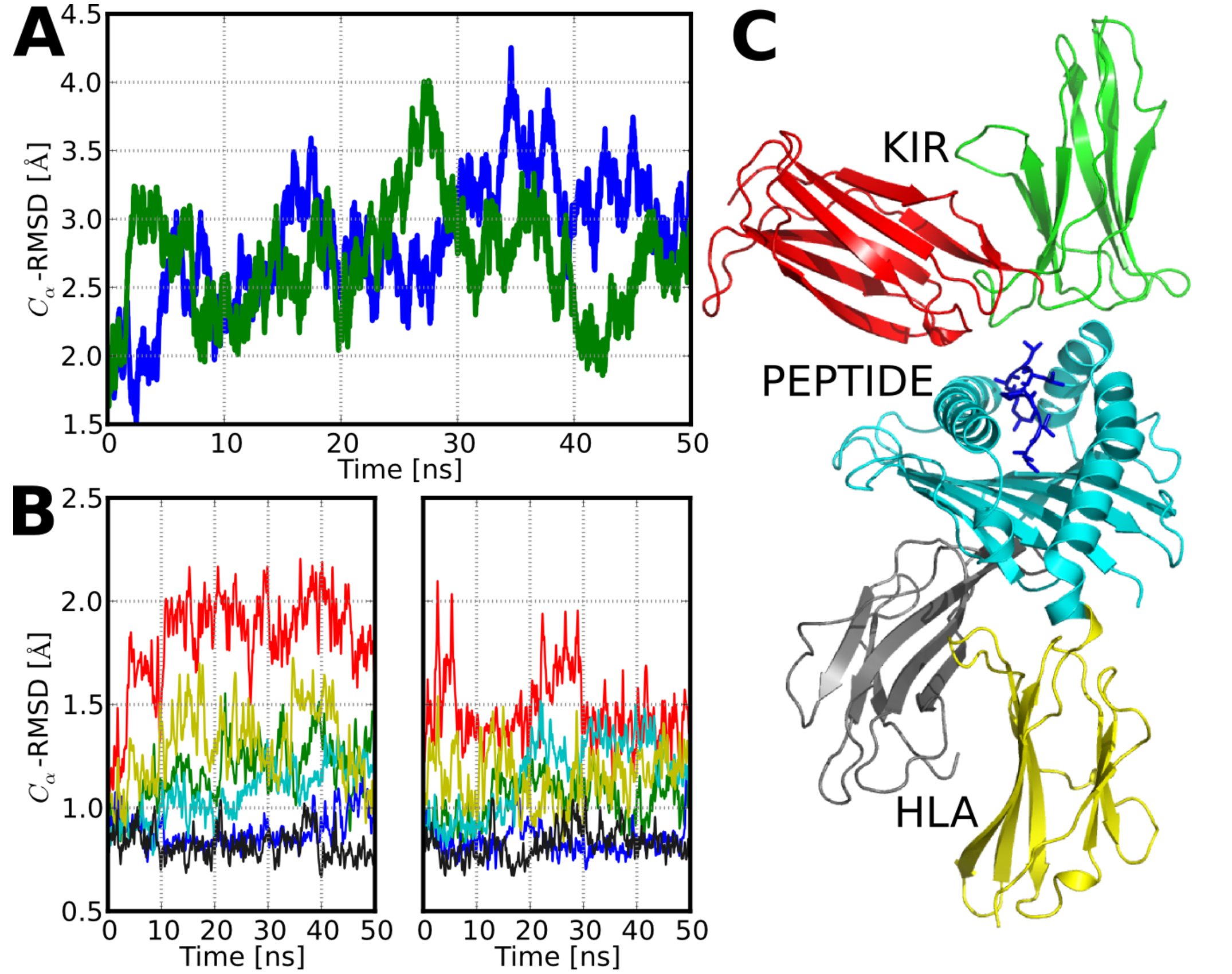

Supplement: S1 Fig — The simulations show a stable 3-D structure during the simulation time, with most of the deformations contained in hinge-like motions. (A) Overall Cα RMSD in the crystal structure versus time for two independent simulations. (B) Cα RMSD in the crystal structure on a per-domain basis. (C) Color labeling for the different domains considered. (TIF) [file pmed.1001900.s001.tif]

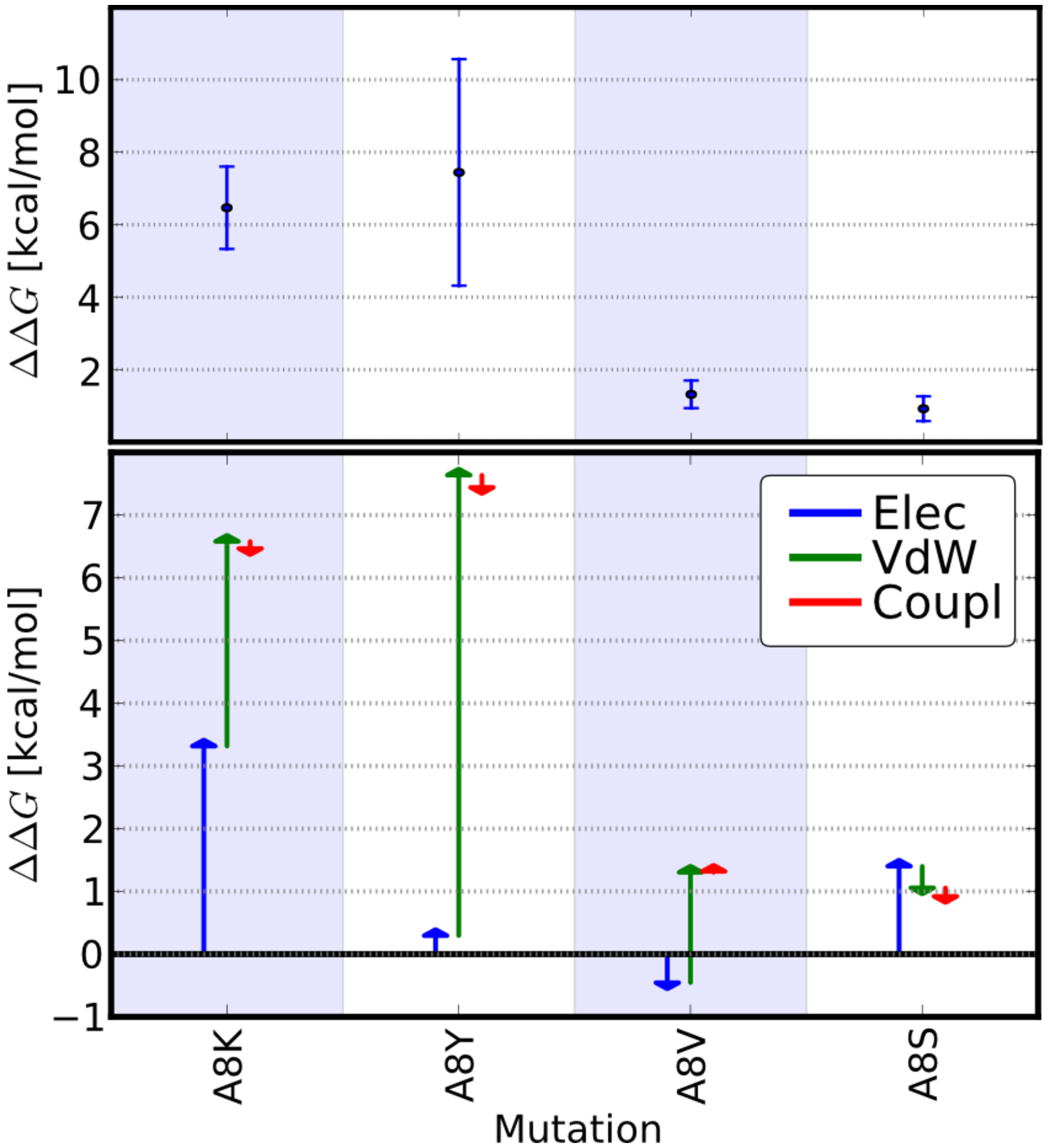

Supplement: S2 Fig — Mutation of the p8 alanine to lysine or tyrosine results in a severe loss of binding affinity (in excess of 6 kcal/mol), with a large component of entropic contribution. Mutation to valine or serine results in more modest destabilization of the complex. (A) Binding free energy changes of each mutant with respect to GAL. (B) Decomposition of the free energy difference in the van der Waals and electrostatic contributions. The limited space around position 8 of the peptide induces preference for smaller peptides, reducing binding affinity for larger or bulky amino acids. (TIF) [file pmed.1001900.s002.tif]

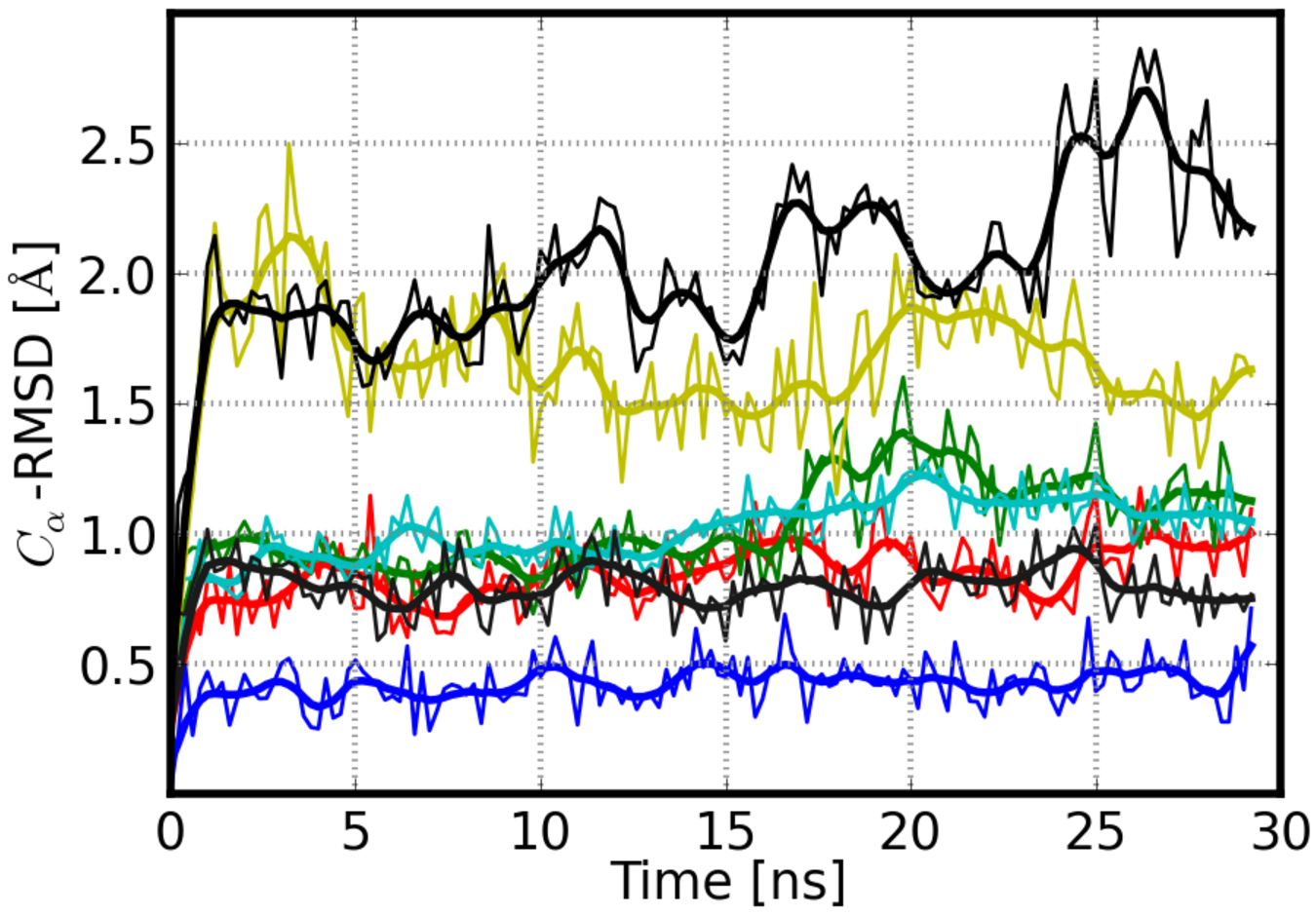

Supplement: S3 Fig — The self-peptide in 1EFX was replaced with the TGag303V (YVL) mutant in a two-step process (see Methods) and simulated for a total of 100 ns. Here, results for a 30-ns trajectory are shown. The black curves show the overall deformation, and the other colors follow the scheme described in S1 Fig. Upon replacement and equilibration, the system remained stable at the interface, displaying small variations mainly contained in the α3 domain of the HLA molecule. (TIF) [file pmed.1001900.s003.tif]

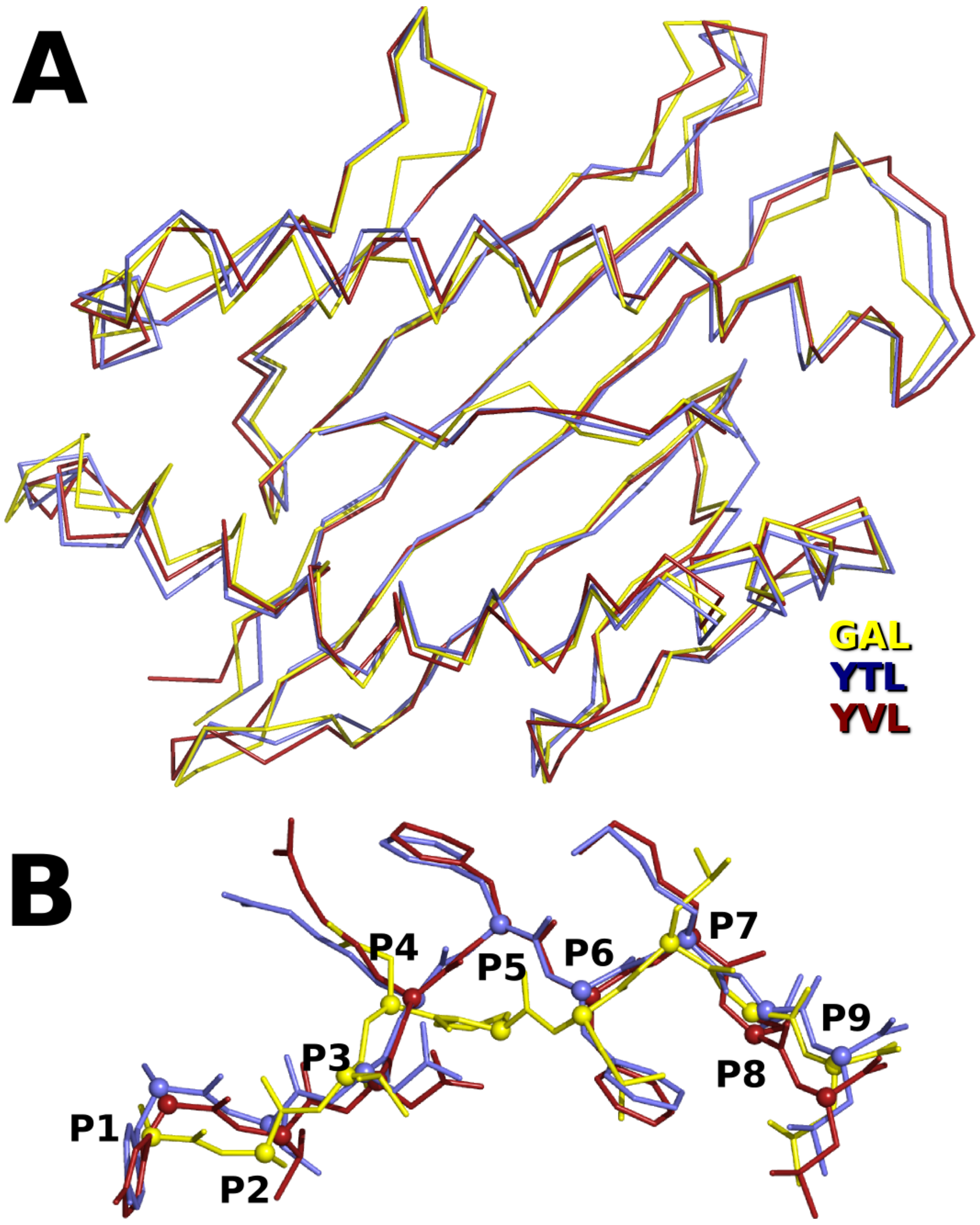

Supplement: S4 Fig — (A)The peptide binding groove is largely insensitive to the identity of the peptide. A superposition of the self-peptide (GAL), the viral wild-type sequence (YTL), and a selected mutant (YVL) is shown. (B)The peptide recognition is provided by hydrogen bonds in the two termini (not shown) but allows for large variability in the central region of the peptide (residues P4, P5, and P6). (TIF) [file pmed.1001900.s004.tif]

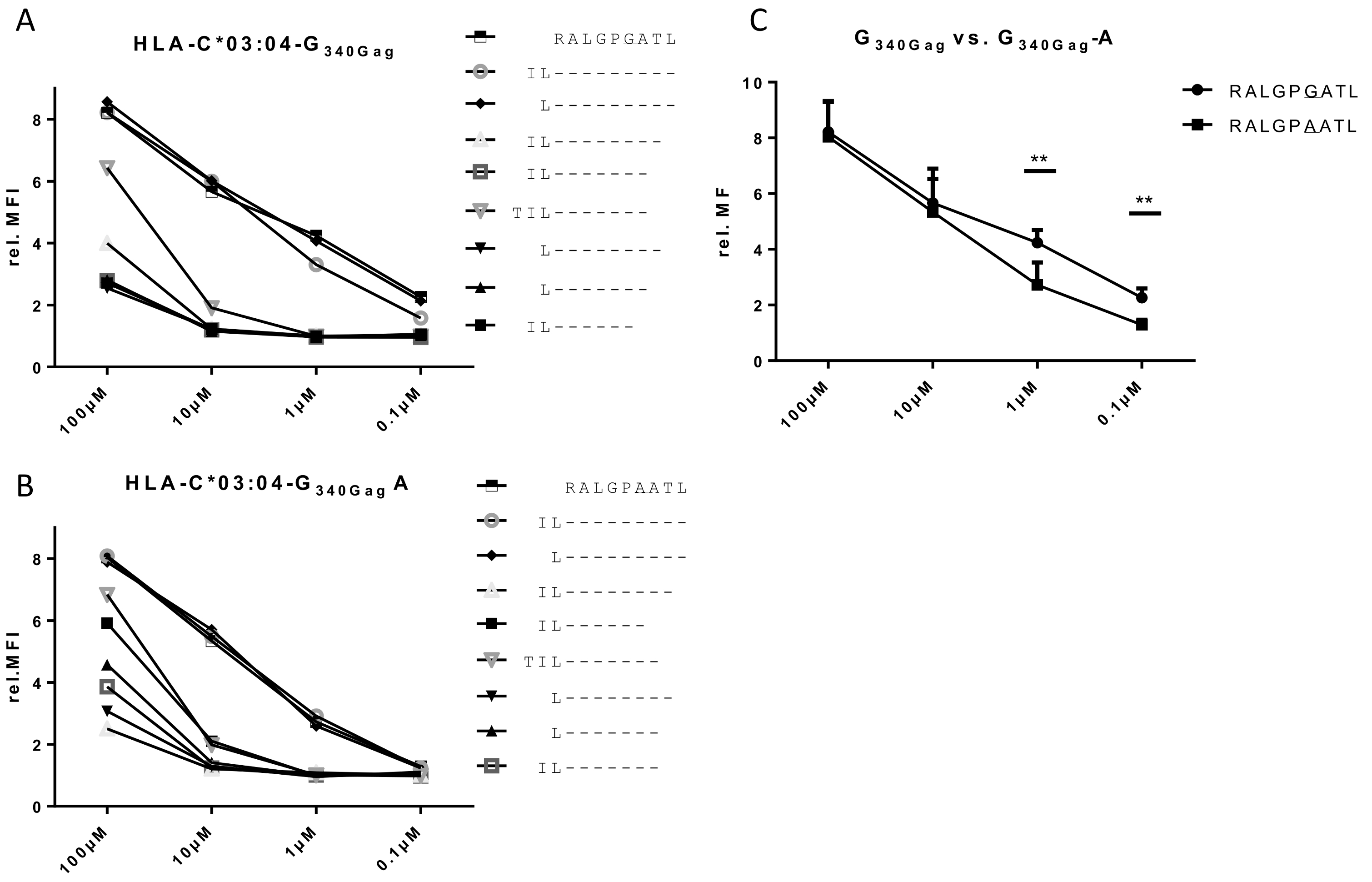

Supplement: S5 Fig — The optimal epitope was determined by the level of HLA-C*03:04 stabilization on TAP-blocked 721.221-ICP47-C*03:04 target cells pulsed with peptides of differing length containing wild-type amino acid G (A) or variant amino acid A (B) at position Gag340. The HLA stabilization assay was performed with decreasing concentrations until non-saturating levels of peptide labeling were reached. We identified RALGPGATL and RALGPAATL as the optimal HLA-C*03:04-restricted epitopes. (C) The wild-type peptide RALGPGATL stabilized HLA-C*03:04 expression on 721.221-ICP47-C*03:04 cells significantly better than the variant epitope RALGPAATL at non-saturating concentrations of 1 μm (G [mean 4.24 ± 0.46 SD] to A [mean 2.72 ± 0.81 SD), p = 0.006) and 0.1 μM (G [mean 2.26 ± 0.39 SD] to A [mean 1.29 ± 0.19 SD], p = 0.008) as measured by paired, two-tailed t-test. HLA-C*03:04 surface expression was determined flow cytometrically by staining with the anti-pan-HLA antibody W6/32 (n = 3). (TIF) [file pmed.1001900.s005.tif]
