# Supplementary material for: Selection of an HLA-C*03:04-Restricted HIV-1 p24 Gag Sequence Variant Is Associated with Viral Escape from KIR2DL3+ Natural Killer Cells: Data from an Observational Cohort in South Africa
Source: PLoS Med. 2015 Nov 17;12(11):e1001900. doi: 10.1371/journal.pmed.1001900 (PMC4648589; doi:10.1371/journal.pmed.1001900)
Supplement: S1 Text — (PDF) [file pmed.1001900.s006.pdf]

Supplemental Material

Details of the computational modelling

The computational models were based on the crystal structure with PDB-id 1EFX. This crystal structure contains two KIR molecules in complex with HLA-C*03 and the peptide GAVDPLLAL (termed GAL). The system, consisting of one KIR molecule bound to the peptide-loaded HLA molecule, was solvated in a 77.5 A x 121.2 A x 78.3 A water box, charge-neutralized with counter ions, and further ionized to match *in vivo* physiological salt concentration of 150mM NaCl for a total of about 60.000 atoms. Once solvated, energy minimization was followed with 5ns of NPT simulation at 1 atm and 310K to generate an equilibrated structure.

Two independent simulations were carried for 50ns to assess stability of the complex under the simulation conditions, and the results can be seen in Figure S1. Once the stability of the system was tested, the final structure of one of the simulations was used as starting structure for the subsequent FEP runs. Each FEP consisted in the alchemic transformation from WT to the target mutant by using a FEP parameter λ, ranging from 0 (WT) to 1 (mutant). The perturbation was carried in windows defined by the following λ values: 0.00, 0.00001, 0.0001, 0.001, 0.01, 0.05, 0.1, 0.2, 0.3, 0.4, 0.5, 0.6, 0.7, 0.8, 0.9, 0.95, 0.99, 0.999, 0.9999, 0.99999, and 1.0. In each window, 4000 steps of equilibration are followed with 200000 steps of perturbation to calculate the variations on energy in function of λ. To obtain converged results for the $\Delta\Delta G$ of binding upon mutation, an average of 9 independent runs were carried for each mutation in each state (bound and free). The resulting differences can be seen in figure S2. The results are consistent with the experimental data reported here for the positive and negative controls, along with previously reported studies of other mutants (Boyington et al., 2000). Mutations GAVDPLLKL (A8K) and GAVDPLLYL (A8Y) abrogate KIR binding by imposing a large free energy penalty (> 6 kcal/mol). Similarly, smaller residues such as Serine and Valine in position 8 of the peptide allow for binding of the NK receptor, but still have a negative effect in the binding affinity of the complex. Taken together, the previous results validate the methodology used, and encourage its extension to the study of the viral peptide.

Since the convergence of a FEP calculation is highly dependent on an appropriate description of the binding mode, and guided by the experimental results suggesting that the binding affinity of the $T_{Gag303}V$ variant is higher than that of the wild type, the configuration used to perform the perturbations was obtained in a two steps process: first, the self-peptide from 1EFX was replaced with the $T_{Gag303}V$ mutant, and after 50ns of equilibration the wild-type sequence was introduced by changing the valine into a threonine, followed by 3 simulations totaling over 100ns. In the first part ($T_{Gag303}V$), the peptide/HLA/KIR complex remained stable during the simulation, with the largest deformation being the twisting of the α3 sub-domain of the HLA molecule (far from the binding interface). After simulating the $T_{Gag303}V$ variant, the peptide position 8 was mutated into a threonine, and further simulated to assure a stabilized initial structure, Figure S3.

The secondary structure and packing of the HLA around the binding groove are largely insensitive to the identity of the peptide (Figure S4a). The peptide is securely anchored in the binding pocket by a dense network of hydrogen bonds around its two termini. However, the amino acids in positions 4-7 are exposed to the solvent, allowing for drastic variations in the overall conformation of the peptide without affecting much the binding pocket. Moreover, the conformation of the HLA and peptide are almost identical for the case of the wild type and $T_{Gag303}V$ mutant, with only a slight displacement of the residue p9 (Figure S4b).

Once the wild type model of the viral peptide was obtained, this structure was used as starting point for the free energy perturbation studies. The changes in free energy of binding upon mutation were calculated by using a thermodynamic cycle, which involves the calculation of free energy differences due to mutations for a bound and free system. Consequently, several independent FEP calculations (9 in average) were used for each system (bound and free) for each mutation.

While KIR2DL2 and KIR2DL3 differ only in four amino acids located far from the binding interface, the same process was repeated using the latter receptor, and the results obtained did not show significant differences.

All simulations used CHARMM22 force field interacting with TIP3P water molecules. The MD and FEP were carried using the NAMD package optimized to run in Blue Gene

computing architecture and a time step of 2 fs. Long-range electrostatic interactions were treated using the particle-mesh Ewald summation method, while van der Waals interactions were calculated using a smooth cut-off of 1.2 nm.
